# Transcriptomic profiling reveals candidate allelopathic genes in rice responsible for interactions with barnyardgrass

**DOI:** 10.3389/fpls.2023.1104951

**Published:** 2023-02-17

**Authors:** Most. Humaira Sultana, Md. Alamin, Jie Qiu, Longjiang Fan, Chuyu Ye

**Affiliations:** ^1^ Institutue of Crop Science and Institute of Bioinformatics, College of Agriculture and Biotechnology, Zhejiang University, Hangzhou, China; ^2^ Department of Biology, School of Life Sciences, Southern University of Science and Technology, Shenzhen, China

**Keywords:** transcriptome, allelopathy, barnyardgrasss, *Echinochola crus-galli*, rice, rice and barnyardgrasss interaction

## Abstract

*Echinochloa crus-galli* (barnyardgrass) is one of the most damaging weeds in rice fields worldwide. Allelopathy has been considered a possible application for weed management. Thus understanding its molecular mechanisms is important for rice production. This study generated transcriptomes from rice under mono- and co-culture with barnyardgrass at two-time points to identify the candidate genes controlling allelopathic interactions between rice and barnyardgrass. A total of 5,684 differentially expressed genes (DEGs) were detected, amongst which 388 genes were transcription factors. These DEGs include genes associated with momilactone and phenolic acid biosynthesis, which play critical roles in allelopathy. Additionally, we found significantly more DEGs at 3 hours than at 3 days, suggesting a quick allelopathic response in rice. Up-regulated DEGs involve diverse biological processes, such as response to stimulus and pathways related to phenylpropanoid and secondary metabolites biosynthesis. Down-regulated DEGs were involved in developmental processes, indicating a balance between growth and stress response to allelopathy from barnyardgrass. Comparison of DEGs between rice and barnyardgrass shows few common genes, suggesting different mechanisms underlying allelopathic interaction in these two species. Our results offer an important basis for identifying of candidate genes responsible for rice and barnyardgrass interactions and contribute valuable resources for revealing its molecular mechanisms.

## Introduction

Rice (*Oryza sativa*) has played a vital role in human nutrition and culture for over 10,000 years. It is one of the major staple crops worldwide, and more than fifty percent of the world’s population consumes it (Khush, 2005). Asian countries grow and eat more than 90% of the rice in the world (Khush, 2005). In the middle of this century, the global population will reach 9 billion if it continues to grow at current projections (Godfray et al., 2010). To meet the demand caused by the increasing world population, income, and consumption, we will have to increase rice production by 70% by 2050 (Varshney et al., 2011; Alamin et al., 2018). We need rice varieties with higher potential and higher yield stability to meet the challenges of manufacturing more rice from suitable lands (Khush, 2005). Much promise for developing new varieties is due to recent advancements in rice genomics.

Weeds are unacceptable and redundant plants that adversely influence human benefit by using land and water resources (Rao, 2000). Rice yield is decreased by weeds that compete with crops for nutrients, moisture, and light. Various factors further mediate yields, such as crop cultivars, weed variety, and the relative density of the crop and weeds (Rezaeieh et al., 2015). Damage caused by weeds is greater than that caused by pests (Oerke, 2006). Research has shown that weeds reduced crop production equivalent to $95 billion per annum worldwide (Lundkvist and Verwijst, 2011). Barnyardgrass (*Echinochloa crus-galli*) is a universally harmful weed within the grass family (Guo et al., 2017). It is a typical and devastating weed in paddy fields (Xuan et al., 2006). It has been reported that barnyardgrass made complexity in 61 countries and a minimum of 36 different crops (Xuan et al., 2006). Research showed that 35% of rice yield globally loses due to interactions with barnyardgrass (Oerke and Dehne, 2004).

Allelopathy is the biological incident in which one organism influences another organism’s growth, survival, or reproduction by releasing biochemical-termed allelochemicals (Cheng and Cheng, 2015). Secondary metabolites are the main component of allelochemicals, which are discharged into the atmosphere throughout the usual pathways (Cheng and Cheng, 2015). The allelochemicals reduce plant development due to the changes in plant growth controllers or phytohormones (Cheng and Cheng, 2015). Plants’ most general and universal secondary metabolites are flavonoids (Du et al., 2010). Plant growth, advancement and adjustment to stress are influenced by flavonoids, which are created by a wing of the phenylpropanoid pathway (Shah and Smith, 2020). Auxin transport is regulated by flavonoids, resulting in the development of a special tissue and, subsequently, the whole plant influenced (Singh et al., 2021; Ahammed and Li, 2022). Terpenoids are architecturally diverse and play a significant role in different defense mechanisms in the plant (Dudareva et al., 2004; Cheng et al., 2007). Phenylpropanoid is a key component of plant-particularized metabolism. It acts as the main biochemical modulator of plant communication with insects and germs, acting with opposing attractive and offensive functions in protective phytoalexin reactions to contagion and herbivory (Ferrer et al., 2008). Pigmentation, defense against UV photodamage, morphological strength by polymeric lignin and diverse antimicrobial phytoalexins supported by phenylpropanoid (Ferrer et al., 2008). Lignin is the collective term for a significant collection of aromatic polymers, and this polymerase is stored mainly in the secondary cell wall (Vanholme et al., 2010). In lignin biosynthesis, CAD is one of the first enzymes, and many CAD homologs have been isolated from diverse plant species, including rice (Vanholme et al., 2010).

Several secondary metabolites, including fatty acids, indoles, momilactones, phenolics and terpenes, were discovered as major allelochoemicals in rice plants, which suppress the growth of Barnyardgrass (Khanh et al., 2005). Recently, much more interest has been given to allelopathy research owing to its possible application for weed controlling and crop production (Khanh et al., 2007). Rice allelopathy has been used for controlling barnyardgrass in many studies (Xuan et al., 2006). Moreover, flavonoids, diterpenoids, and other biochemicals have been determined as effective allelochemicals from different parts of rice plants (Kong, 2007). Thus, rice allelopathy would accelerate the defense mechanism by the chemically mediated interchange between rice and Barnyardgrass plants (Kato-Noguchi, 2011). Many studies have been conducted using the molecular approach for the biosynthesis of momilactones and phenolic acids and their allelopathic impacts on barnyardgrass. Momilactones A played a vital role in rice protection against mycological pathogens (Mennan et al., 2012) and momilactone B is an important allelochemical in the rice-barnyardgrass interaction (Kato-Noguchi and Peters, 2013). Two gene clusters have been identified in the synthesis of the momilactones (Xu et al., 2012; Kato-Noguchi and Peters, 2013). It was proposed that the biosynthesis path of phenolic synthesis might be part of the acute allelopathic apparatuses for rice. This is because the PAL (phenylalanine ammonia-lyase) was more susceptible to allelopathic rice, PI312777, than non-allelopathic rice (Fang et al., 2010; Fang et al., 2013; Zhang et al., 2018). Other study demonstrated that the improved expression level of several genes, including *COMT*, *C4H*, *PAL*, and *F5H*, were associated with phenolic compounds of rice, resulting in a reverse impact on barnyardgrass (He et al., 2012).

Biological research has been changing rapidly due to current advances in genomic sequencing technologies, which significantly affect crop improvement (Edwards and Batley, 2010; Mochida and Shinozaki, 2010). Transcriptomics research has been done using the next-generation sequencing (NGS) supported RNA-Seq technique is a robust tool for exploring the function of genes in various tissues and varied environments (Chen et al., 2011). Zhang et al. (2010) reported that transcriptome analysis is very important for discovering genotypic and environmental interactions. However, genomics and bioinformatics information is still limited for weeds affecting modern crops, in particular, the involvement of transcription factors remains largely unknown (Duke and Gressel, 2010). Much progress has been made in NGS technology in recent years, yet few studies have focused on the biological mechanisms of the rice and barnyardgrass interaction.

In this study, RNA-seq was utilized to discover the genes as well as their functional relationship for controlling rice and barnyardgrass interactions at different time points. To improve the genomic sources for weeds, we sequenced the transcriptomes of rice co-cultured with barnyardgrass using the Illumina platform. In the present study, clustering of putative functional categories was carried out with the Gene Ontology (GO) structure, alignment into route utilization with the Kyoto Encyclopedia of Genes and Genomes (KEGG) databank, and visualization of profiling data sets within the context of existing knowledge for the functional investigation of DEGs utilizing the MapMan software. This study provides valuable genetic information regarding barnyardgrass and rice interaction and is beneficial for practical weed regulation.

## Materials and methods

### Plant materials and growth conditions

The allelopathic experiment between rice (PI312777) and barnyardgrass (STB08) was conducted according to methods previously described (Guo et al., 2017; Sultana et al., 2019). In detail, the adapted transmit seeding in the agar method (Navarez and Olofsdotter, 1996) was applied to examine the allelopathic connections among rice and barnyardgrass. Ten PI312777 sprouted seeds were initially transmitted to a pot (10 cm basis dimension) compacted with 50 mL of 0.5% agar medium, and STB08 sprouted seeds were relocated to a dish with ddH_2_0. The PI312777 seeds were organized in three lines with a 3-4-3 formation, and ten STB08 sprouted seeds were then relocated to the identical dish after 5 days, and five seeds were grownup between rows of PI312777 spores (**Supplementary Figure S1**). The PI312777 and STB08 plants were co-cultivated for 3h and 3d in a SAFE chamber (Ningbo, China) at 30°C in light (14 h) environments, 20°C in darkness (10 h) environments, and 75% moisture. The controls comprised of PI312777 alone grown in a container. The first sample (whole plant) and second sample (whole plant) were collected after 3h and 3d of treatment, rinsed five times with ddH_2_0, instantly frozen in liquid N_2_, and kept in RNase-free tubes at −80°C for RNA separation. For each analysis, ten plants from one pot were pooled. Three replicates for both control and treatment were used in this study.

### RNA extraction, cDNA library preparation, and illumina sequencing

Total RNAs were extracted employing the RNeasy Plant Mini Kit (Qiagen) as stated by the manufacturer’s guidelines. The first-strand cDNA was produced by a First Strand cDNA Synthesis kit (TaKaRa, Dalian, China). Illumina RNA-Seq libraries for PI312777 at 3h and 3d time points were organized and sequenced on a HiSeq 4000 technique complying with the producer’s guidelines.

### Data filtering, read mapping, and gene quantification

Data were purified earlier in the downstream investigation to reduce data noise. Reads with adaptors, low-quality reads, and reads with more than 10% unknown bases were removed. NGS QC Toolkit version v2.3.3 was utilized to attribute and purify of the sequencing data with the standard setting (Patel and Jain, 2012). Clean reads, the remaining reads after filtering, were saved as a FASTQ data format (Cock et al., 2010). Bowtie (Langmead et al., 2009) and TopHat2 (Kim et al., 2013) were utilized to map clean reads to reference genes and to the reference genome of the Nipponbare subspecies of rice (MSU7.0, http://rice.plantbiology.msu.edu/), respectively. After alignment, the Cufflinks package was applied to calculate the number of reads that were mapped to each gene within the gene model annotation file. The subsequent alignment files were then provided to Cuffdiff in the Cufflinks package to determine every gene’s FPKM (fragments per kilobase per million reads) value.

### Differentially expressed genes analysis and annotation

The Cuffdiff method (Trapnell et al., 2012) was used for identifying the DEGs (differentially expressed genes). DEGs were selected with *p*-value < 0.05 regarded as DEGs. The Gene Ontology **(**GO) enrichment was executed utilizing agriGO v2.0 (Tian et al., 2017) with Singular Enrichment Analysis (SEA), *Oryza sativa* japonica was chosen as the species and MSU7.0 gene ID (TIGR) as a reference background. The Fisher test with Yekutieli (FDR under dependency) Multi-test adjustment approach was used based on the significance level 0.05. The KO-Based Annotation System (KOBAS) (Xie et al., 2011) was used for getting a summary of the gene pathway network for KEGG analysis. The MapMan (version 3.6.0RC1) and pathways were downloaded from the MapMan Site (http://mapman.gabipd.org/mapmanstore). The mapping info of rice genes (osa_MSU_v7_mapping.txt.tar.gz) was imported to the MapMan to get a more standard overview of deviations in gene expression. To measure common genes between all samples of a two-time points experiment, we used jvenn (http://jvenn.toulouse.inra.fr/app/index.html), an integrative tool for comparing lists with Venn Diagrams which offers statistic charts founded on input data. Circos (Krzywinski et al., 2009) version-0.69-6 was used to visualize details of a chromosomal view of DEGs. The PlantTFDB database (http://planttfdb.cbi.pku.edu.cn/prediction.php) was used to identify transcription factors of DEGs.

### Time-series expression profile

STEM (Short Time-series Expression Miner) was used to investigate short time-series expression data (Ernst and Bar-Joseph, 2006). It uses an innovative clustering process to distinguish between actual and arbitrary shape and group genes by allocating them to expression profiles. A profile is considered important if the total of genes allocated to it exceeds the number of genes predicted to happen by chance. The number of significant genes allocated to each profile against the projected number was calculated and modified for incorrect detection rate at the arrangement of a gene. The limit for the STEM clustering procedure was fixed to 50 for the “maximum number of model profiles” and the “maximum unit variation in model profiles across time points” was set to two to accept clustering at a realistic number of probable model profiles.

### Identification of homolog genes

To identify the rice DEGs homologs to barnyardgrass (Guo et al., 2017) and previously identified allelochemical genes (Amb and Ahluwalia, 2016), we created a protein database by using *O. sativa* MSU7 protein sequences. BLASTP was then utilized to scan homologous genes in the protein database considering thresholds: 10^−10^ (E-value).

### Gene expression validation using qRT-PCR

The Trizol (Invitrogen, Carlsbad, CA, USA) method was used for RNA extraction based on the company’s protocol. cDNA preparation and qRT-PCR (quantitative real-time PCR) was performed according to a previous study (Sultana et al., 2019). The protocol for the qRT-PCR was as follows: 95°C for 30 s, 45 cycles at 95°C for 10 s for denaturation, annealing at 60°C for 10 s, and extension at 72°C for 20 s. Three biological repeats were implemented for every treatment. Relative expression levels were estimated following the process of (Livak and Schmittgen, 2001). We have calculated the qRT-PCR log2 (FC) values based on the calculation of the log2 (normalized ratio values) from the mean values of three replicates for each gene and compared them with RNA-Seq log2 (FC) values. The rice *actin* gene was utilized as a control, and the primers for the gene used in qRT-PCR are provided in **Supplementary Table S1**.

## Results

### Summary of RNA-seq data

The rice cultivar PI312777 (known to have high allelopathic potential) was cultivated independently (mono-cultured) or co-cultured with barnyardgrass to find potential genes that participated in allelopathy of rice against barnyardgrass. Rice transcriptomic data were then generated at two-time points (3h and 3d) with three biological replicates in control (mono-cultured) and treatment (co-cultured with barnyardgrass) conditions using RNA-Seq. There were 25.97 million (3h) and 25.48 million (3d) raw reads in twelve samples (**Table 1**). The numbers of total clean reads were 256 million (3h) and 250 million (3d) in twelve samples (**Table 1**). The overall average mapping rates were 75.12% (**Table 1**).

**Table 1:** Mapping results of RNA sequencing reads.

Time points	Sample ID	Raw bases	Clean bases (%)	Raw reads	Clean reads (%)	Mapped reads (%)	Multiple alignments (%)
**3h**	3h_C1	5968809900	5796177274 (98.64%)	39792066	39175940 (98.45%)	28011714 (71.5%)	878439 (3.1%)
	3h_C2	6417289200	6262043338 (98.81%)	42781928	42250364 (98.76%)	31823432 (75.3%)	1397216 (4.4%)
	3h_C3	5768741400	5605836673 (98.7%)	38458276	37863546 (98.45%)	27263894 (72.2%)	845754 (3.1%)
	3h_T1	6209253300	6033434398 (98.64%)	41395022	40777296 (98.51%)	29353016 (71.9%)	1202374 (4.1%)
	3h_T2	8228647200	7929442588 (97.98%)	54857648	53950196 (98.35%)	37421744 (69.4%)	760703 (2%)
	3h_T3	6370019700	6113475248 (97.97%)	42466798	41599722 (97.96%)	28632334 (68.8%)	978825 (3.5%)
**3d**	3d_C1	5942038800	5846652549 (98.39%)	39792066	38977948 (98.4%)	29779939 (76.4%)	1044610 (3.5%)
	3d_C2	5929839300	5840730517 (98.49%)	42781928	39058816 (98.8%)	30807236 (78.9%)	708212 (2.3%)
	3d_C3	6627531300	6521318373 (98.39%)	38458276	43387320 (98.2%)	33754145 (77.8%)	1084281 (3.3%)
	3d_T1	7238626200	7123865032 (98.42%)	41395022	47472718 (98.37%)	38364086 (80.8%)	1098492 (2.9%)
	3d_T2	6706004400	6597933758 (98.39%)	54857648	44002982 (98.43%)	34153651 (77.6%)	812104 (2.4%)
	3d_T3	5778936900	5620990114 (97.26%)	42466798	37578398 (97.54%)	30372971 (80.8%)	1018915 (3.4%)

N.B 3h C1 and 3h T1 mean control sample 1 and treatment sample 1, respectively, at a 3-hour time point. 3d C1 and 3d T1 mean control sample 1 and treatment sample 1, respectively, at a 3-day time point. Other sample names indicate a similar way.

### Identification of differentially expressed genes

**Figure 1:**
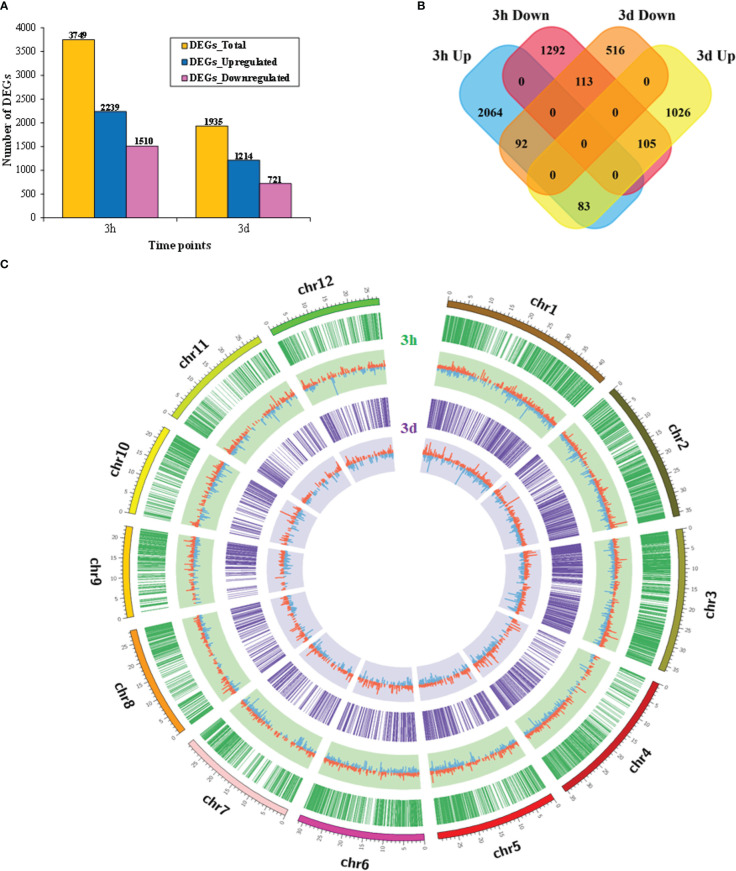
The expression profile of rice differentially expressed genes (DEGs) grown with barnyardgrass. **(A)** Bar plot represents the total number, up-regulated, and down-regulated DEGs at 3h and 3d time points, respectively. **(B)** Venn diagrams represent the numbers of DEGs and the overlaps of sets obtained across the two time points. **(C)** Distribution features of DEGs of the rice genome under rice and barnyardgrass interaction using a Circos plot. Different colors show the sizes of the 12 chromosomes in rice. The green circle and light green color represent the distribution and expression (log2 FC) value of DEGs, respectively, at 3h. The purple circle and light purple color represent the distribution and expression (log2 FC) value of DEGs, respectively, at 3d. Up- and down-regulated genes are shown by red and blue, respectively.

A total of 5,684 differentially expressed genes (DEGs) were identified. Among them, 3,749 (65.96%) and 1,935 (34.04%) DEGs were identified at 3h and 3d time points, respectively. DEGs with up-regulation were more common than those with down-regulation (**Figure 1A**). Moreover, a total of 393 common DEGs were found at the two-time points. Among them, 83 up-regulated and 113 down-regulated genes were common, 92 genes were up-regulated at 3h and down-regulated at 3d, and 105 genes were down-regulated at 3h and up-regulated at 3d (**Figure 1B**). Furthermore, a Circos plot was drawn to show up- and down-regulated gene distribution patterns in chromosomes at 3h and 3d. Results showed that the DEGs were evenly distributed over the 12 chromosomes in rice (**Figure 1C**).

### Identification of allelochemical responsible transcription factors

**Figure 2:**
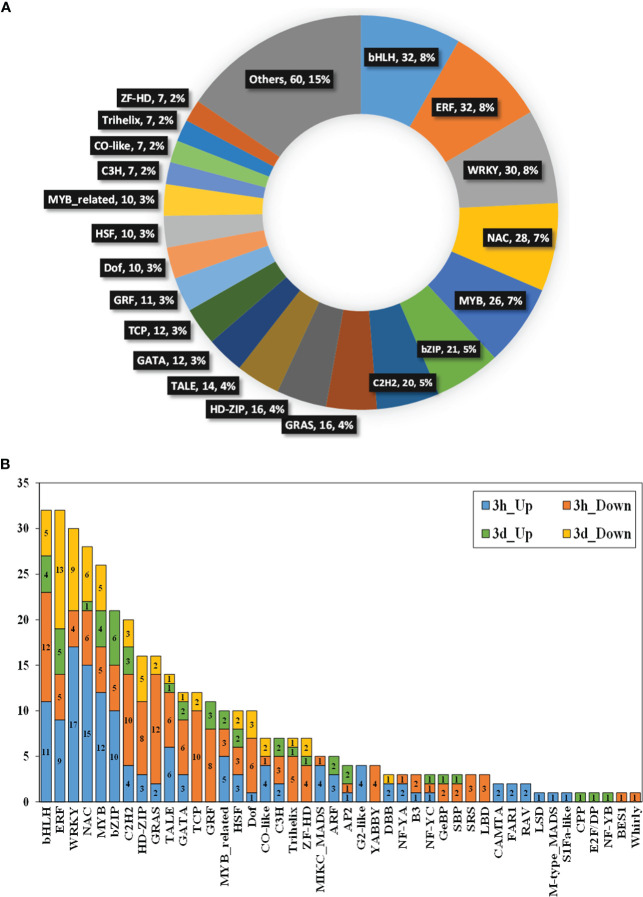
Transcription factors (TFs) analysis of DEGs identified for rice and barnyardgrass interaction. **(A)** Pie chart represent the TFs families with their number and percentage for all DEGs. **(B)** Number of genes associated with different transcription factors at 3h and 3d time points. Different colors represent different transcription factor gene families and the genes number for up-regulated and down-regulated genes for 3h and 3d time points.

The PlantTFDB database was used to identify the transcription factors (TFs) (Jin et al., 2017). A total of 388 TFs from 44 different TFs families were detected to be DEGs, such as bHLH, ERF, WRKY, NAC, MYB, bZIP, C2H2, GRAS, HD-ZIP, TALE, and GATA families (**Figure 1A**, **Supplementary Table S2**). These TFs accounted for more than 50% of the total differentially expressed TFs. More differentially expressed TFs (278 TFs from 41 families) were found at 3h than those (110 TFs from 28 families) at the 3d time point (**Figure 2B**, **Supplementary Table S2**).

### Gene ontology and pathway enrichment analysis of DEGs

**Figure 3:**
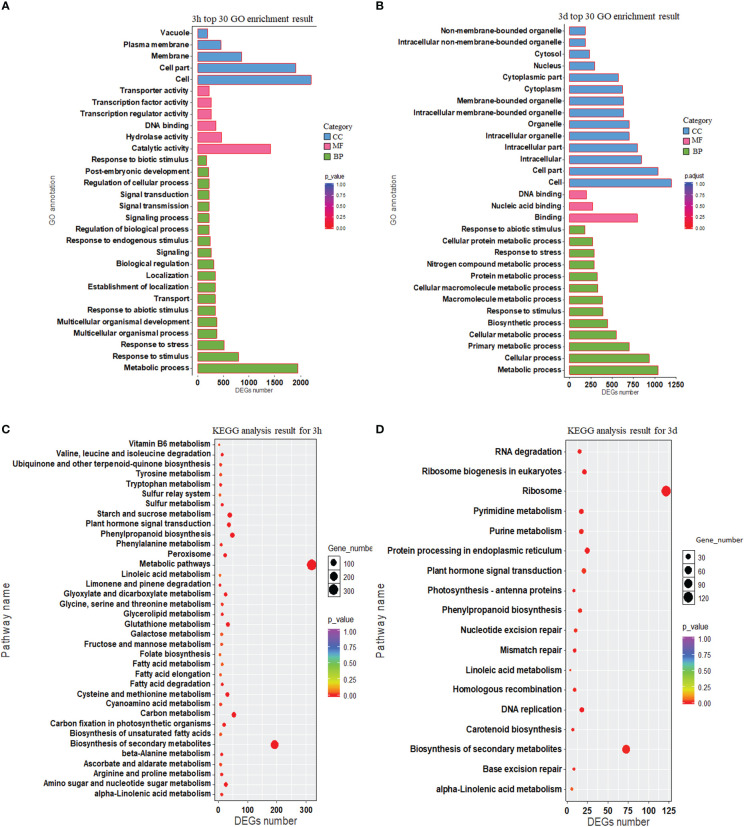
The functional annotation of rice DEGs. **(A, B)** Gene ontology (GO) annotation of DEGs for 3h and 3d. The vertical axis represents functional annotation information and the horizontal axis represents the number of differential genes annotated to the function. **(C, D)** KEGG pathway enrichment presented using a scatter plot for 3h and 3d. The vertical axis and horizontal axis represented by pathway name and DEGs number, respectively. The *p*-value ranges from 0 to 1 and the *p*-value is represented by the color of the dot; the smaller the *p*-value, the closer the color is to red. The relative number of DEGs contained under each function is denoted by the size of the dots.

Gene Ontology (GO) enrichment analysis for DEGs at 3h and 3d was performed (*P* < 0.05; **Supplementary Table S3**). The top 30 GO terms in each time point are given in **Figures 3A, B**. Twelve GO terms were common between the two-time points, which were “response to stimulus”, “metabolic process”, “response to abiotic stimulus”, “response to biotic stimulus”, “response to stress”, “DNA binding”, “extracellular region”, “external encapsulating structure”, “cell wall”, “cell”, “cell part”, and “vacuole”.

Genes typically act together with one another to take part in specific biological activities. KEGG database (Kanehisa et al., 2016) dependent pathway analysis was carried out to investigate biological processes during the interaction of rice and barnyardgrass. The results showed that 1,098 DEGs were involved in the 36 pathways at 3h, and 404 DEGs were involved in 18 pathways at 3d time point (**Figures 3C, D**, **Supplementary Table S4**). Most genes were enriched in “metabolic pathways”, followed by “biosynthesis of secondary metabolites”, “carbon metabolism”, “phenylpropanoid biosynthesis”, “starch and sucrose metabolism”, “plant hormone signal transduction”, “glutathione metabolism and cysteine”, and “methionine metabolism” at 3h (**Figure 3C**, **Supplementary Table S4**). “Ribosome,” “biosynthesis of secondary metabolites,” and “plant hormone signal transduction” were the most enriched KEGG at 3d (**Figure 3D**, **Supplementary Table S4**). The common pathways at the two-time points include “phenylpropanoid biosynthesis” (osa00940), “biosynthesis of secondary metabolites” (osa01110), and “plant hormone signal transduction” (osa04075).

**Figure 4:**
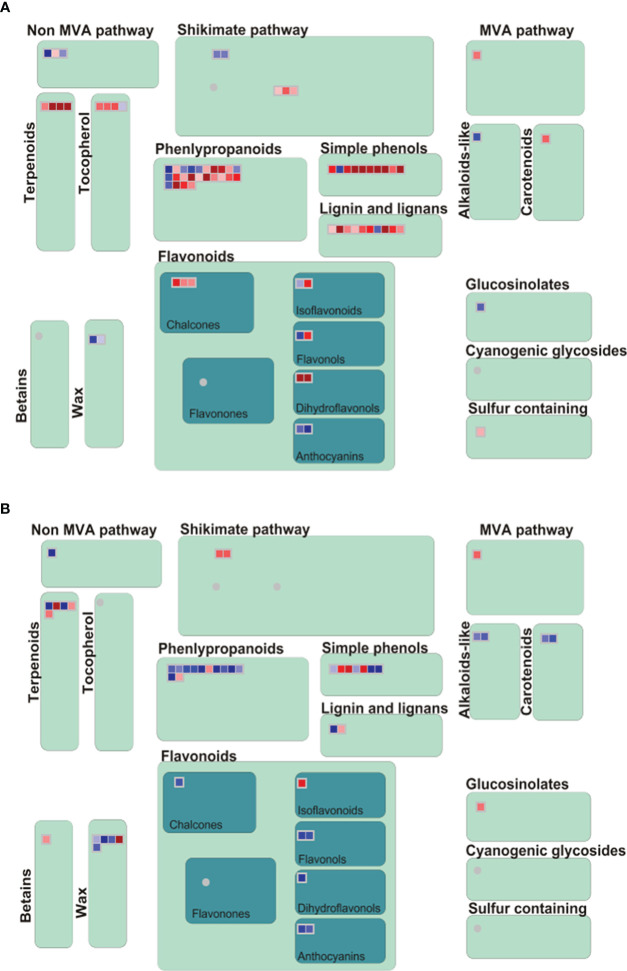
Analyses of secondary metabolism pathway of DEG using MAPMANs. **(A, B)** Secondary metabolism pathway analysis at 3h and 3d. Red and blue boxes indicate up-regulated and down-regulated genes.

**Figure 5:**
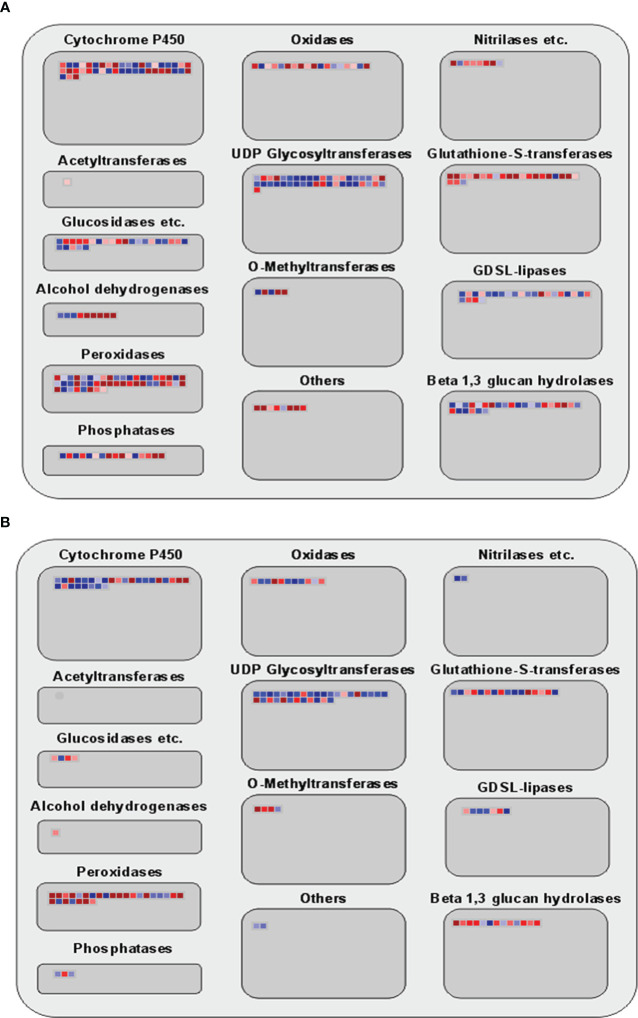
Analyses of enzyme family pathway of DEGs using MAPMAN. **(A, B)** Large enzyme family pathways of 3h and 3d time points. Red and blue boxes indicate up-regulated and down-regulated genes.

Many genes have been identified in this study that are involved in metabolic and enzyme pathways in allelopathy interactions. Therefore, a secondary metabolic and large enzyme pathway of DEGs was investigated using MAPMAN. Results showed that the expression of genes associated with terpenoids, phenylpropanoids, simple phenols, lignin and lignans, and different flavonoid pathways were noticeably up-regulated at 3h (**Figures 4A, B**). Moreover, different enzymes, including Cytochrome P450, UDP glycosyltransferase, glutathione-S-transferases, glucosidases, GDSL-lipases, Beta 1,3 glucan hydrolases, O-methyltransferases, peroxidases, and phosphatase were involved at the two-time points (**Figures 5A, B**).

### Clustering of time-series expression profile

**Figure 6:**
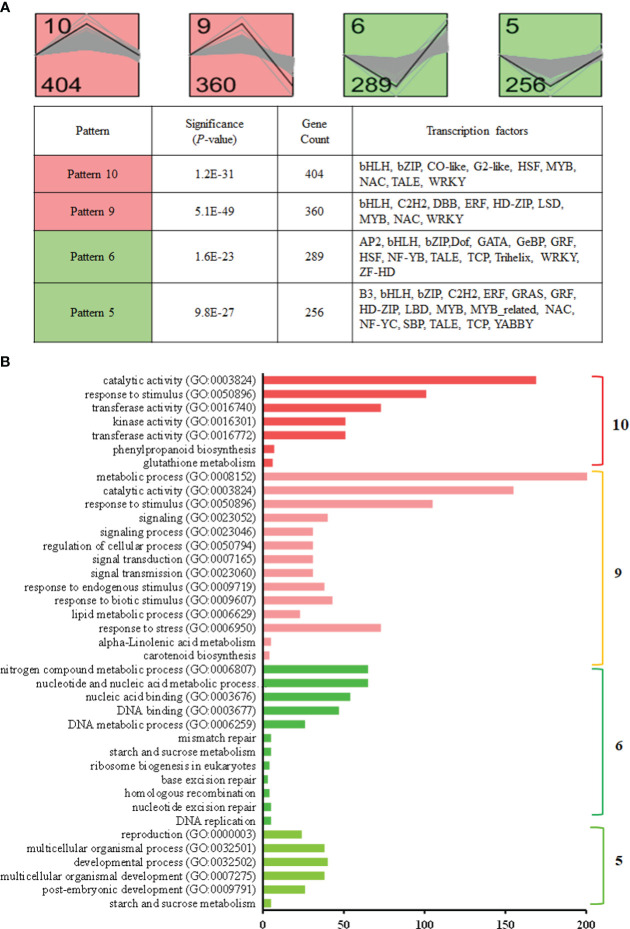
Dynamic change analysis of differentially co-expressed DEGs. **(A)** Protein clusters identified using the STEM algorithm that demonstrates coherent changes during 3h and 3d time points. Top panel: Model profiles based on fold change values over time for 3h and 3d. Significant profiles are highlighted in different colors. The black line indicates the fitted profile; gray lines are individual gene profiles. The top left number indicates the model profile number; the bottom left number indicates the number of genes contained in the profile. Bottom panel: the significance (*p*-value), gene counts, and transcription factors in the four representative gene expression patterns. **(B)** Functional enrichment based on gene patterns for significant profiles.

STEM was used to perform time-series expression profile clustering to exploration for shared temporal expression patterns based on our 5,684 DEGs. We identified four highly significant (*P* < 0.05) major temporal expression profiles that showed consistent expression patterns in rice responsive to barnyardgrass co-cultivation (**Figure 6A**, **Supplementary Table S5**). Generally, four main expression cluster groups were identified.

In Profile 10 (404 genes), most of these genes’ expression was up-regulated at 3h, but not changed at 3d, in rice responsive to barnyardgrass co-cultivation. In this pattern, genes were exclusively enriched in two pathways, namely glutathione metabolism (osa00480) and phenylpropanoid biosynthesis (osa00940) (**Figure 6B**, **Supplementary Table S5**). Profile 9 (360 genes) showed that the genes were up-regulated at 3h, followed by down-regulation at 3d, and the genes were enriched in two pathways, including alpha-Linolenic acid metabolism (osa00592) and carotenoid biosynthesis (osa00906). Additionally, genes in both profiles were enriched in the biological process of “response to stimulus” (**Figure 6B**, **Supplementary Table S5**).

The other two profiles (256 genes in Profile 5 and 289 genes in Profile 6) consist of genes that are decreased at 3h. These genes were enriched in the “nitrogen compound metabolic process” and “development process” for GO terms and starch and sucrose metabolism (osa00500) for the KEGG pathway (**Figure 6B**, **Supplementary Table S5**).

### Comparison of DEGs in rice and barnyardgrass under co-cultivation

Previously, we have obtained transcriptomic data of barnyardgrass co-cultured with rice at 3h (Guo et al., 2017). A total of 4,945 DEGs, including 2,534 up-regulated and 2,411 down-regulated genes, were discovered in barnyardgrass. Here we have identified 396 homologous DEGs; 151 genes are unique (without duplicate) between rice and barnyardgrass at the 3h time point. Using a heat map, we have represented the homologous DEGs’ expression values (**Supplementary Figure S2**). Results clearly showed a distinct expression pattern between rice and barnyardgrass.

### Expression patterns of rice allelochemical genes

**Figure 7:**
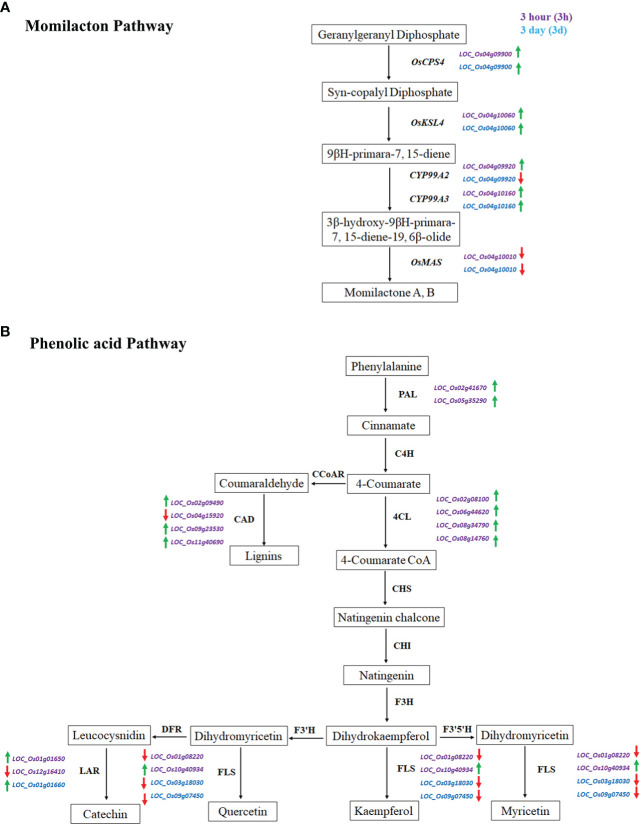
Expression of momilactone and phenolic acid pathways related genes during the rice and barnyardgrass interaction. **(A)** Momilactone pathway and **(B)** Phenolic acid pathway. Up-regulated and down-regulated genes denoted by green and red arrows, respectively.

Two important pathways, namely momilactone- and phenolic acid-related genes, were involved during rice and barnyardgrass interaction. We have investigated five previously identified momilactone genes. Results showed that *OsCPS4* (*LOC_Os04g09900*)*, OsKSL4* (*LOC_Os04g10060*), and *CYP99A3* (*LOC_Os04g10160*) are up-regulated in both 3h and 3d time points. *CYP99A2* (*LOC_Os04g09920*) is up-regulated at 3h but down-regulated at 3d, and *OsMAS* (*LOC_Os04g10010*) is down-regulated at both time points (**Figure 7A**). The expressions of these genes in rice were proposed to respond to the allelopathic interaction between rice and barnyardgrass.

For the phenolic acid pathway, we found two PAL (phenylalanine ammonia-lyase) up-regulated DEGs, *LOC_Os05g35290* and *LOC_Os02g41670*; four CAD (cinnamyl-alcohol dehydrogenase) genes where *LOC_Os04g15920* is down-regulated and *LOC_Os11g40690*, *LOC_Os02g09490*, and *LOC_Os09g23530* up-regulated at DEGs at 3h time points in our analysis. Four up-regulated 4CL (4-coumaroyl CoA ligase) DEGs, *LOC_Os02g08100*, *LOC_Os06g44620*, *LOC_Os08g34790*, and *LOC_Os08g14760* were found at 3h which is an important branch point resulting to the group of flavonoids, lignins, and lignans. A total of seven DEGs were found in myricetin, kaempferol, quercetin, and catechin at both time points (**Figure 7B**). The gene expression results suggest that both momilactone and phenolic acid pathways are related to allelopathy, and it is difficult to pinpoint which is more important.

We have collected 55 genes or clones of previously identified allelopathy-associated genes from different rice varieties (Amb and Ahluwalia, 2016) and used blast for these protein sequences with our identified DEGs’ proteins to identify the rice allelochemical-related genes in this study. A total of 286 homolog DEGs of the putative allelopathy-associated genes were identified at 3h and 3d time points in this study. Among them, we have found significantly more genes at 3h than 3d, i.e., 201 (150 up- and 51 down-regulated) and 85 (62 up- and 23 down-regulated) genes at 3h and 3d, respectively, were identified (**Supplementary Table S6**).

### qRT-PCR analysis results for RNA-Seq data validation

We have randomly used 17 genes to confirm the RNA-Seq data for 3h and 3d time points. The qRT-PCR results suggested that all studied genes displayed similar expression trends to the RNA-Seq results (**Table 2**). As the many common genes (393 genes) were involved in rice and barnyardgrass interactions, we investigated the eight common genes for 3h and 3d time points (**Table 2**). All the genes were up-regulated at 3h, but *LOC_Os12g25490* (O-methyltransferase) and *LOC_Os01g43750* (cytochrome P450) were down-regulated at 3d time point, which is consistent with RNA-Seq results. Three momilactone-related genes showed similar results, i.e., up-regulation at both 3h and 3d. Furthermore, four up-regulated genes involved in phenolic acid pathways at 3h were investigated by qRT-PCR. Similar patterns were observed between RNA-Seq expression results and qRT-PCR results. One gene (*LOC_Os01g01660*) was up-regulated and another gene was down-regulated (*LOC_Os03g18030*) for the two phenolic acid pathway-related genes at 3d (as compared with control) for the rice and barnyardgrass interaction (**Table 2**).

**Table 2:** RNA-Seq data validation using qRT-PCR.

	3h time point			3d time point		
	Gene Name	RNA_seq log_2_(FC)	qRT-PCR log_2_(FC)	Gene Name	RNA_seq log_2_(FC)	qRT-PCR log_2_(FC)
Common genes in 3h and 3d time points	LOC_Os03g13210	2.06	2.56	LOC_Os03g13210	0.66	1.32
	LOC_Os12g25490	1.47	1.65	LOC_Os12g25490	-0.56	-0.24
	LOC_Os03g56090	1.02	1.63	LOC_Os03g56090	0.86	0.98
	LOC_Os05g20930	0.9	1.3	LOC_Os05g20930	1.95	1.75
	LOC_Os09g35030	1.02	2.04	LOC_Os09g35030	0.73	1.63
	LOC_Os10g17260	1.14	1.57	LOC_Os10g17260	0.59	0.25
	LOC_Os06g37300	0.85	1.17	LOC_Os06g37300	1.26	1.18
	LOC_Os01g43750	0.92	2.85	LOC_Os01g43750	-0.93	-2.81
Momilactone pathway related genes	LOC_Os04g10160	1.06	2.71	LOC_Os04g10160	0.62	0.4
	LOC_Os04g10060	1.64	2.07	LOC_Os04g10060	0.99	0.61
	LOC_Os04g09900	1.27	1.67	LOC_Os04g09900	0.12	0.19
Phenolic acid pathway related genes	LOC_Os05g35290	0.39	0.45	LOC_Os01g01660	0.87	0.48
	LOC_Os02g41670	1.13	1.19	LOC_Os03g18030	-0.91	-1.1
	LOC_Os11g40690	1.72	1.55			
	LOC_Os01g01650	0.86	0.76			

## Discussion

Major challenges in maximizing crop yields involve weeds, and allelopathy is a favorable technique for weed regulation worldwide (Fang et al., 2013). Transcriptomics research has been done using the NGS supported RNA-Seq technique—a reliable tool for exploring the function of genes in various tissues and environments (Chen et al., 2011). In this study, we have performed RNA-Seq analyses to explore the changes in the transcriptome of rice co-cultured with barnyardgrass at the seedling stage. We have identified a total of 5684 DEGs at two-time points, with significantly more DEGs at 3 hours than 3 days, suggesting a quick allelopathic response in rice (**Figure 1**). This is also true for known allelopathy-related genes, for which significantly more genes at 3h (201) than 3d (85) were DEGs (Supplementary Table S6). We also found 84 common up-regulated genes for the two time-periods, suggesting that these genes may be continuously leading the allelopathic response to barnyardgrass.

Research showed that the transcription factor was regulating the gene expression by controlling transcription beginning speed, where appropriate gene expression for each organism is very significant for their enlargement and maturity (Corrêa et al., 2008). TFs have a significant function for controlling plant propagation, maturation, and react to unfavorable situation conditions, including drought, chill, salinity, and high temperature (Zhai et al., 2013). Studies showed that different types of TFs play essential roles in various functional pathways such as bHLP involved in phytochrome signaling activity (Zhang et al., 2009), ERF involved diverse responses to environmental stimuli (Nakano et al., 2006), NAC involved in various mechanisms including developmental process (Nuruzzaman et al., 2010), and MYB participated in the control of anthocyanin biosynthesis (Ambawat et al., 2013). We have identified a total of 388 genes were related to 44 different TFs families, where most of them were allocated in the bHLH, ERF, WRKY, NAC, MYB, and bZIP (**Figure 2A**). Specifically, more TF-encoding genes were down-regulated (207 TFs) than up-regulated (181 TFs) in rice and barnyardgrass interaction (**Figure 2B**, **Supplementary Table S2**). From the TFs analysis results, we assumed that these TFs might be involved in rice and barnyardgrass interaction.

We found DEGs enriched in stress response-associated GO terms, such as “response to stimulus”, “response to biotic stimulus” and “response to stress” which was expected as allelopathy interactions are a kind of stress to plants. Four significant temporal expression profiles showing consistent expression patterns in rice responsive to barnyardgrass co-cultivation have been identified, for which two profiles (Profiles 5 and 6) consist of genes that are decreased at 3h (**Figure 6**, **Supplementary Table S5**). Interestingly, these down-regulated genes were enriched in the “nitrogen compound metabolic process” and “development process” for GO terms and starch and sucrose metabolism (osa00500) for the KEGG pathway. This suggests a balance between growth and stress (allelopathy) response in rice.

Genes related to phenylpropanoid metabolism might increase the synthesis and discharge of allelochemicals and inhibit weeds (Fang et al., 2010). A recent study demonstrated that the DEGs were connected with phenylpropanoid biosynthesis, phenylalanine metabolism, and tyrosine biosynthesis in both allelopathic and non-allelopathic rice against control (Zhang et al., 2018). The KEGG pathway investigation exhibited that most genes were involved in “metabolic pathways”, followed by “biosynthesis of secondary metabolites”, “carbon metabolism”, “phenylpropanoid biosynthesis”, “starch and sucrose metabolism”, “plant hormone signal transduction”, and “glutathione metabolism” in this study (**Figures 3C, D**). KEGG results indicated that various pathways, including “phenylpropanoid biosynthesis”, might play a crucial function in controlling rice and barnyardgrass interactions.

Previous studies described the flavones, fatty acids, phenolic acids, steroids, and terpenoids are the rice allelochemicals (Kong et al., 2004; Seal et al., 2004; Macías et al., 2006; He et al., 2012; Kato-Noguchi and Peters, 2013; Zhang et al., 2018). Cytochrome P450 (P450s) are extensive in plant genomes, and the function of P450s are distributed in diverse biochemical pathway to create primary and secondary metabolites, including lipids, phenylpropanoids, terpenoids, and alkaloids (Mizutani and Ohta, 2010). Research showed that P450 CYP93G2 was the main enzyme for establishing *C*-glycosylflavones from flavanones in rice (Du et al., 2010). Glutathione S-transferase (GST), a broad category of main defense enzymes, is essential in protecting cells from various abiotic and biotic stress, including xenobiotic and heavy-metal perniciousness, oxidative stress, and pathogen assault in plants (Soranzo et al., 2004; Peng et al., 2023). A recent study showed that GST is essential for glutathione-bisphenol A mating and use of exogenous dopamine to plant enhanced glutathione levels resulting expands organic contaminant detoxification and stress resistance of cucumber seedlings (Ahammed and Li, 2023). Several studies demonstrated that the rice allelopathy is an inherited attribute connected with the molecular mediation of secondary metabolic pathways (Bi et al., 2007; Song et al., 2008; Fang et al., 2009). MapMan software was used along through secondary metabolism and enzyme pathway analysis in this study. Our results did show that genes involved in various secondary metabolic pathways, including terpenoids, phenylpropanoids, simple phenols, lignin and lignans, and different flavonoids pathways, were noticeably up-regulated in this study (**Figure 4**). Also, different enzymes, including cytochrome P450, glutathione-S-transferases, and glucosidases, were involved at 3h and 3d time points (**Figure 5**). These findings indicated that allelopathic interaction is a complicated process between plants instead of a single critical pathway. Additionally, we compared the DEGs between rice and barnyardgrass and showed that very few genes were common. Particularly, expression patterns of common DEGs are not similar. These results demonstrated different mechanisms underlying the allelopathy interaction between the two species.

In summary, RNA-Seq data used to detect the crucial DEGs, gene functional analysis, KEGG pathways analysis, transcription factors related to rice allelopathy, and allelopathy related genes and their molecular mechanisms that impact of weeds on rice in this study. We supposed that the momilactone pathway might be more sensitive against barnyardgrass than other phenolic acid pathways from the expression pattern of rice allelochemical genes. The outcomes of the present study might provide more straight proof and evidence for the forthcoming study on the interaction between rice and barnyardgrass. We believe that this study could provide a valuable genetic resource for rice and barnyardgrass associated candidate allelopathy genes and should be useful for controlling weeds, which would outcome in the development of agriculture. However, the detailed roles of the identified genes related to allelopathy controlling weeds could be needed for further functional analysis by genetic approaches involved in rice and barnyardgrass interaction. Also, this study was inspected in the lab, which unable to scrutinize the interaction of rice and barnyardgrass in the natural field condition. Moreover, genome-wide association study (GWAS) is a well-know and widely used method for dissecting complex traits in plants (Yang et al., 2014; Alamin et al., 2022). GWAS could be utilized to identify the important variants associated with the interaction of rice and barnyardgrass. Furthermore, candidate genes associated with the interaction of rice and barnyardgrass could be identified by the expression of quantitative trait loci analysis.

## Data availability statement

The data presented in the study are deposited in the BioProject repository, accession number PRJNA645506.

## Author contributions

Conceptualization: CY and LF. Formal analysis: MS and MA. Validation: MS, MA, and CY. Writing-original draft: MS and MA. Writing-review and editing: CY, JQ, and LF. Funding acquisition: CY and LF. All authors contributed to the article and approved the submitted version.
