# Supplementary material for: Transcriptomic profiling reveals candidate allelopathic genes in rice responsible for interactions with barnyardgrass
Source: Front Plant Sci. 2023 Feb 17;14:1104951. doi: 10.3389/fpls.2023.1104951 (PMC9982016; doi:10.3389/fpls.2023.1104951)
Supplement: Supplementary file 1 [file DataSheet_1.docx]

**Supplementary materials**

**Supplementary Figure**

**Supplementary Figure S1.** The placement map of Rice (PI3I2777) seeds, *E. crus-galli* seeds and their interaction.


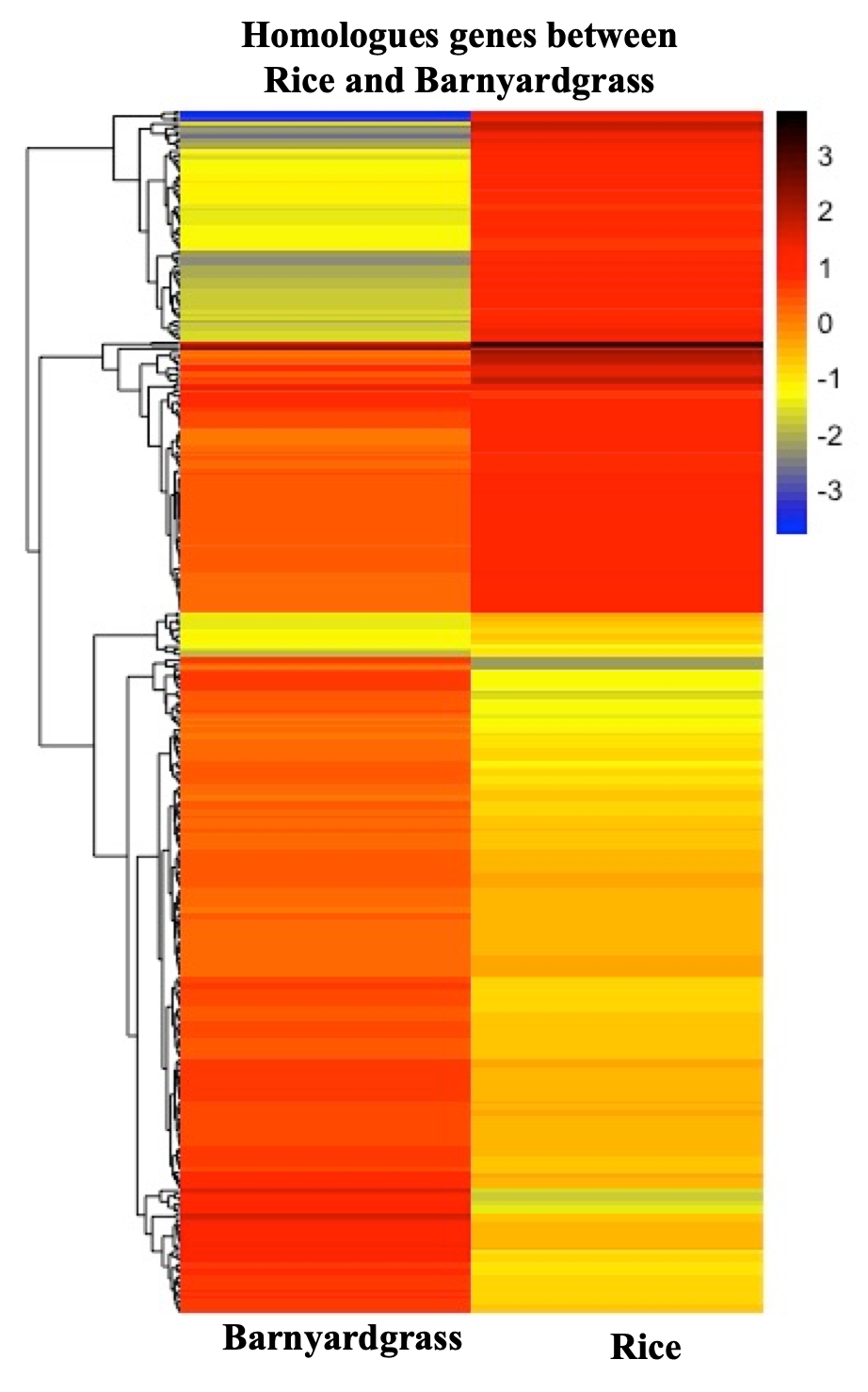


**Supplementary Figure S2. Homologues genes analysis between rice and barnyardgrass.**  Heat map for barnyardgrass and rice homologues genes at 3h time point.

**Supplementary Tables**

**Supplementary Table S1. Primers used in qRT-PCR in this study.**

| Name | Annotation | Forward primer (5´–3´) | Reverse primer (5´–3´) |
| --- | --- | --- | --- |
| Rice actin | Rice actin | GTGGTCGCCCCTCCTGAAAG | GGCTTAGCATTCTTGGGTCCG |
| LOC_Os03g13210 | peroxidase precursor | TCGGTGAGAGGGTACGAAGT | CGAATCGAAAGGGCTAGGCA |
| LOC_Os12g25490 | O-methyltransferase | CATTCAGAGCAACGGGCAACA | AGATGCCAGAGAGAGTGAGCA |
| LOC_Os03g56090 | MYB family transcription factor | CTCGGAGGAGGAAGACAGGA | CAAGTGTAATTGGCACCGGC |
| LOC_Os05g20930 | ZOS5-07 - C2H2 zinc finger protein | CTGCCTTGGGCAAACAAGTC | GCCAATCGAACAGCACCAAG |
| LOC_Os04g10160 | cytochrome P450 | GGATGGCATGGCAGACTACA | GGTGACGACGAAGTCCATGT |
| LOC_Os04g10060 | ent-kaurene synthase, chloroplast precursor | GGTTGCTGGTCAGGTAGCTT | GCACGGCATGTTTTTGGTCT |
| LOC_Os05g35290 | phenylalanine ammonia-lyase | GACCACCTCACCCACAAGC | TCCTGCTTCGGCTTCATCAG |
| LOC_Os02g41670 | phenylalanine ammonia-lyase | CTTGGAAGGCAGCTCGTACA | TCTGAGGGCCAAGCCATTG |
| LOC_Os11g40690 | dehydrogenase | GTGGCAACTGTGATGCTTGG | AGTTCTCGCAGTCATGGTCG |
| LOC_Os01g01660 | isoflavone reductase | GATCCCGTTGAACATCGTGC | GTCATCGACGGTGGTGTACT |
| LOC_Os03g18030 | leucoanthocyanidin dioxygenase | AGGGGCCTCCAGGTGAAGAA | GCGTTGCTCAGCACCTGTATC |
| LOC_Os01g01650 | isoflavone reductase homolog IRL | TGCAACTACTTCGCCGGATT | GGCACGTACACTCTCTCCAG |
| LOC_Os09g35030 | dehydration-responsive element-binding protein | GACGTCCTGAGTGACATGGG | AGTAGCTCCAGAGTGGGACG |
| LOC_Os10g17260 | cytochrome P450 | GTGGATGTCAAGGGTGCTGA | GGCAGTCATCAGTGTGACCA |
| LOC_Os01g43750 | cytochrome P450 72A1 | CACCAGCCGTAACCTTCACT | CTCCCCCAGATGTTTGGGTC |
| LOC_Os04g09900 | ent-kaurene synthase, chloroplast precursor | GGGTTCTTGCCCAGGAGAAT | GTCTCAGGTGAGCAGTAGGC |
| LOC_Os06g37300 | cytochrome P450 | GAGGCTGACTGGAGGGACTA | ACATGGTCCATTCGGTGGTC |

**Supplementary Table S2. Summary of transcription factors (TFs) analysis for DEGs.**

| **Transcriptome Factor family** | **3h_Up** | **3h_Down** | **3d_Up** | **3d_Down** | **Total** |
| --- | --- | --- | --- | --- | --- |
| bHLH | 11 | 12 | 4 | 5 | 32 |
| ERF | 9 | 5 | 5 | 13 | 32 |
| WRKY | 17 | 4 |  | 9 | 30 |
| NAC | 15 | 6 | 1 | 6 | 28 |
| MYB | 12 | 5 | 4 | 5 | 26 |
| bZIP | 10 | 5 | 6 |  | 21 |
| C2H2 | 4 | 10 | 3 | 3 | 20 |
| HD-ZIP | 3 | 8 |  | 5 | 16 |
| GRAS | 2 | 12 |  | 2 | 16 |
| TALE | 6 | 6 | 1 | 1 | 14 |
| GATA | 3 | 6 | 2 | 1 | 12 |
| TCP |  | 10 |  | 2 | 12 |
| GRF |  | 8 | 3 |  | 11 |
| MYB_related | 5 | 3 | 2 |  | 10 |
| HSF | 3 | 3 | 2 | 2 | 10 |
| Dof | 1 | 6 |  | 3 | 10 |
| CO-like | 4 | 1 |  | 2 | 7 |
| C3H | 2 | 3 | 2 |  | 7 |
| Trihelix |  | 5 | 1 | 1 | 7 |
| ZF-HD |  | 4 | 1 | 2 | 7 |
| MIKC_MADS | 4 | 1 |  |  | 5 |
| ARF | 3 |  | 2 |  | 5 |
| G2-like | 4 |  |  |  | 4 |
| AP2 | 1 | 1 | 2 |  | 4 |
| YABBY |  | 4 |  |  | 4 |
| DBB | 2 |  |  | 1 | 3 |
| NF-YA | 2 | 1 |  |  | 3 |
| B3 | 1 | 2 |  |  | 3 |
| NF-YC | 1 | 1 | 1 |  | 3 |
| GeBP |  | 2 | 1 |  | 3 |
| LBD |  | 3 |  |  | 3 |
| SBP |  | 2 | 1 |  | 3 |
| SRS |  | 3 |  |  | 3 |
| CAMTA | 2 |  |  |  | 2 |
| FAR1 | 2 |  |  |  | 2 |
| RAV | 2 |  |  |  | 2 |
| LSD | 1 |  |  |  | 1 |
| M-type_MADS | 1 |  |  |  | 1 |
| S1Fa-like | 1 |  |  |  | 1 |
| BES1 |  | 1 |  |  | 1 |
| CPP |  |  | 1 |  | 1 |
| E2F/DP |  |  | 1 |  | 1 |
| NF-YB |  |  | 1 |  | 1 |
| Whirly |  | 1 |  |  | 1 |
|  |  |  |  |  | Sum= 388 |

**Supplementary Table S3. GO enrichment for DEGs at 3h and 3d time points.**

| **Significant GO terms for all DEGs at 3h time point** | | | | | |
| --- | --- | --- | --- | --- | --- |
| Ontology | GO term | Description | Number in input list | Number in BG/Ref | p-value |
| BP | GO:0050896 | response to stimulus | 794 | 5265 | 3.9E-06 |
| BP | GO:0008152 | metabolic process | 1947 | 14183 | 9.6E-05 |
| BP | GO:0009628 | response to abiotic stimulus | 347 | 2194 | 1.9E-04 |
| BP | GO:0009719 | response to endogenous stimulus | 241 | 1491 | 6.7E-04 |
| BP | GO:0009607 | response to biotic stimulus | 180 | 1076 | 8.3E-04 |
| BP | GO:0005975 | carbohydrate metabolic process | 161 | 972 | 2.2E-03 |
| BP | GO:0006950 | response to stress | 515 | 3618 | 1.5E-02 |
| BP | GO:0007165 | signal transduction | 223 | 1464 | 1.1E-02 |
| BP | GO:0050794 | regulation of cellular process | 223 | 1464 | 1.1E-02 |
| BP | GO:0023046 | signaling process | 224 | 1481 | 1.4E-02 |
| BP | GO:0023060 | signal transmission | 224 | 1481 | 1.4E-02 |
| BP | GO:0019748 | secondary metabolic process | 81 | 488 | 2.3E-02 |
| BP | GO:0009791 | post-embryonic development | 213 | 1441 | 3.4E-02 |
| BP | GO:0023052 | signaling | 269 | 1849 | 3.4E-02 |
| BP | GO:0065007 | biological regulation | 312 | 2165 | 3.4E-02 |
| BP | GO:0006810 | transport | 340 | 2374 | 3.5E-02 |
| BP | GO:0051234 | establishment of localization | 340 | 2374 | 3.5E-02 |
| BP | GO:0051179 | localization | 340 | 2374 | 3.5E-02 |
| BP | GO:0007275 | multicellular organismal development | 369 | 2591 | 3.7E-02 |
| BP | GO:0050789 | regulation of biological process | 231 | 1588 | 4.7E-02 |
| BP | GO:0032501 | multicellular organismal process | 373 | 2636 | 4.6E-02 |
| MF | GO:0003824 | catalytic activity | 1415 | 9920 | 5.3E-06 |
| MF | GO:0016787 | hydrolase activity | 466 | 3112 | 1.0E-03 |
| MF | GO:0005215 | transporter activity | 226 | 1457 | 5.5E-03 |
| MF | GO:0030528 | transcription regulator activity | 269 | 1773 | 6.9E-03 |
| MF | GO:0003700 | transcription factor activity | 269 | 1773 | 6.9E-03 |
| MF | GO:0003677 | DNA binding | 355 | 2434 | 1.5E-02 |
| CC | GO:0005576 | extracellular region | 132 | 559 | 2.0E-09 |
| CC | GO:0030312 | external encapsulating structure | 173 | 953 | 3.2E-05 |
| CC | GO:0005618 | cell wall | 171 | 943 | 3.7E-05 |
| CC | GO:0005623 | cell | 2201 | 16308 | 8.0E-04 |
| CC | GO:0016020 | membrane | 855 | 5995 | 1.2E-03 |
| CC | GO:0044464 | cell part | 1904 | 14141 | 6.6E-03 |
| CC | GO:0005886 | plasma membrane | 449 | 3116 | 1.2E-02 |
| CC | GO:0005773 | vacuole | 190 | 1250 | 2.0E-02 |
| **Significant GO terms for all DEGs at 3d time point** | | | | | |
| BP | GO:0034645 | cellular macromolecule biosynthetic process | 142 | 664 | 6.9E-30 |
| BP | GO:0009059 | macromolecule biosynthetic process | 142 | 664 | 6.9E-30 |
| BP | GO:0044249 | cellular biosynthetic process | 142 | 664 | 6.9E-30 |
| BP | GO:0006412 | translation | 142 | 664 | 6.9E-30 |
| BP | GO:0010467 | gene expression | 152 | 791 | 1.3E-27 |
| BP | GO:0006259 | DNA metabolic process | 66 | 434 | 7.3E-09 |
| BP | GO:0009058 | biosynthetic process | 450 | 5311 | 3.7E-08 |
| BP | GO:0008152 | metabolic process | 1043 | 14183 | 1.4E-06 |
| BP | GO:0007049 | cell cycle | 49 | 352 | 5.6E-06 |
| BP | GO:0044260 | cellular macromolecule metabolic process | 333 | 3953 | 6.7E-06 |
| BP | GO:0043170 | macromolecule metabolic process | 387 | 4902 | 2.2E-04 |
| BP | GO:0006950 | response to stress | 290 | 3618 | 7.1E-04 |
| BP | GO:0009628 | response to abiotic stimulus | 182 | 2194 | 2.0E-03 |
| BP | GO:0044238 | primary metabolic process | 697 | 9565 | 2.0E-03 |
| BP | GO:0044237 | cellular metabolic process | 549 | 7497 | 5.8E-03 |
| BP | GO:0044267 | cellular protein metabolic process | 275 | 3573 | 7.5E-03 |
| BP | GO:0009607 | response to biotic stimulus | 94 | 1076 | 7.1E-03 |
| BP | GO:0050896 | response to stimulus | 392 | 5265 | 9.4E-03 |
| BP | GO:0009987 | cellular process | 937 | 13328 | 1.0E-02 |
| BP | GO:0006807 | nitrogen compound metabolic process | 293 | 3898 | 1.7E-02 |
| BP | GO:0006139 | nucleobase, nucleoside, nucleotide and nucleic acid metabolic process | 293 | 3898 | 1.7E-02 |
| BP | GO:0016043 | cellular component organization | 118 | 1451 | 2.0E-02 |
| BP | GO:0019538 | protein metabolic process | 328 | 4462 | 3.1E-02 |
| MF | GO:0005198 | structural molecule activity | 127 | 511 | 4.4E-32 |
| MF | GO:0016818 | hydrolase activity, acting on acid anhydrides, in phosphorus-containing anhydrides | 19 | 85 | 2.1E-05 |
| MF | GO:0016817 | hydrolase activity, acting on acid anhydrides | 19 | 85 | 2.1E-05 |
| MF | GO:0003774 | motor activity | 19 | 85 | 2.1E-05 |
| MF | GO:0016462 | pyrophosphatase activity | 19 | 85 | 2.1E-05 |
| MF | GO:0017111 | nucleoside-triphosphatase activity | 19 | 85 | 2.1E-05 |
| MF | GO:0003676 | nucleic acid binding | 275 | 3349 | 2.5E-04 |
| MF | GO:0003677 | DNA binding | 198 | 2434 | 3.0E-03 |
| MF | GO:0003723 | RNA binding | 56 | 573 | 4.9E-03 |
| MF | GO:0005488 | binding | 794 | 11178 | 9.9E-03 |
| MF | GO:0008135 | translation factor activity, nucleic acid binding | 15 | 133 | 4.4E-02 |
| CC | GO:0030529 | ribonucleoprotein complex | 133 | 476 | 5.1E-38 |
| CC | GO:0005840 | ribosome | 133 | 476 | 5.1E-38 |
| CC | GO:0032991 | macromolecular complex | 133 | 476 | 5.1E-38 |
| CC | GO:0043232 | intracellular non-membrane-bounded organelle | 187 | 918 | 7.5E-37 |
| CC | GO:0043228 | non-membrane-bounded organelle | 187 | 918 | 7.5E-37 |
| CC | GO:0005730 | nucleolus | 89 | 363 | 2.0E-22 |
| CC | GO:0031974 | membrane-enclosed lumen | 96 | 516 | 5.5E-17 |
| CC | GO:0031981 | nuclear lumen | 96 | 516 | 5.5E-17 |
| CC | GO:0043233 | organelle lumen | 96 | 516 | 5.5E-17 |
| CC | GO:0070013 | intracellular organelle lumen | 96 | 516 | 5.5E-17 |
| CC | GO:0044428 | nuclear part | 100 | 582 | 1.2E-15 |
| CC | GO:0044446 | intracellular organelle part | 100 | 582 | 1.2E-15 |
| CC | GO:0044422 | organelle part | 100 | 582 | 1.2E-15 |
| CC | GO:0030312 | external encapsulating structure | 127 | 953 | 3.4E-12 |
| CC | GO:0005618 | cell wall | 125 | 943 | 6.9E-12 |
| CC | GO:0005829 | cytosol | 239 | 2287 | 2.4E-11 |
| CC | GO:0005634 | nucleus | 294 | 3020 | 1.2E-10 |
| CC | GO:0005622 | intracellular | 842 | 10775 | 2.1E-09 |
| CC | GO:0043229 | intracellular organelle | 699 | 8737 | 5.9E-09 |
| CC | GO:0043226 | organelle | 699 | 8737 | 5.9E-09 |
| CC | GO:0005623 | cell | 1195 | 16308 | 3.1E-08 |
| CC | GO:0044424 | intracellular part | 798 | 10327 | 8.5E-08 |
| CC | GO:0044464 | cell part | 1042 | 14141 | 9.6E-07 |
| CC | GO:0005576 | extracellular region | 65 | 559 | 3.7E-05 |
| CC | GO:0005773 | vacuole | 120 | 1250 | 1.0E-04 |
| CC | GO:0043231 | intracellular membrane-bounded organelle | 638 | 8465 | 1.3E-04 |
| CC | GO:0043227 | membrane-bounded organelle | 638 | 8465 | 1.3E-04 |
| CC | GO:0005783 | endoplasmic reticulum | 59 | 562 | 9.0E-04 |
| CC | GO:0044444 | cytoplasmic part | 575 | 7709 | 9.6E-04 |
| CC | GO:0005737 | cytoplasm | 621 | 8386 | 1.0E-03 |

**Supplementary Table S4. KEGG pathway analysis for DEGs.**

| **KEGG analysis result for all DEGs at 3 hour time point** | | | | |
| --- | --- | --- | --- | --- |
| **Pathways_name** | **ID** | **Input number** | **Background number** | **P-Value** |
| Metabolic pathways | osa01100 | 319 | 1573 | 7.4E-19 |
| Biosynthesis of secondary metabolites | osa01110 | 192 | 848 | 1.6E-15 |
| Phenylpropanoid biosynthesis | osa00940 | 48 | 125 | 1.4E-10 |
| Glutathione metabolism | osa00480 | 34 | 73 | 1.4E-09 |
| Cysteine and methionine metabolism | osa00270 | 32 | 96 | 1.9E-06 |
| Glyoxylate and dicarboxylate metabolism | osa00630 | 25 | 63 | 2.1E-06 |
| Carbon metabolism | osa01200 | 53 | 235 | 2.9E-05 |
| Starch and sucrose metabolism | osa00500 | 39 | 159 | 6.9E-05 |
| Sulfur metabolism | osa00920 | 15 | 35 | 1.1E-04 |
| Peroxisome | osa04146 | 23 | 80 | 3.2E-04 |
| Amino sugar and nucleotide sugar metabolism | osa00520 | 27 | 111 | 9.3E-04 |
| Valine, leucine and isoleucine degradation | osa00280 | 14 | 40 | 9.8E-04 |
| Fatty acid degradation | osa00071 | 14 | 41 | 1.2E-03 |
| beta-Alanine metabolism | osa00410 | 12 | 32 | 1.4E-03 |
| Plant hormone signal transduction | osa04075 | 38 | 184 | 1.5E-03 |
| Arginine and proline metabolism | osa00330 | 13 | 40 | 2.5E-03 |
| Carbon fixation in photosynthetic organisms | osa00710 | 19 | 73 | 2.7E-03 |
| Limonene and pinene degradation | osa00903 | 6 | 9 | 2.7E-03 |
| Glycerolipid metabolism | osa00561 | 14 | 46 | 2.9E-03 |
| Tryptophan metabolism | osa00380 | 9 | 23 | 4.3E-03 |
| alpha-Linolenic acid metabolism | osa00592 | 12 | 39 | 5.2E-03 |
| Phenylalanine metabolism | osa00360 | 11 | 35 | 6.5E-03 |
| Glycine, serine and threonine metabolism | osa00260 | 14 | 57 | 1.4E-02 |
| Ubiquinone and other terpenoid-quinone biosynthesis | osa00130 | 10 | 35 | 1.6E-02 |
| Cyanoamino acid metabolism | osa00460 | 10 | 36 | 1.8E-02 |
| Ascorbate and aldarate metabolism | osa00053 | 10 | 37 | 2.1E-02 |
| Tyrosine metabolism | osa00350 | 10 | 38 | 2.4E-02 |
| Vitamin B6 metabolism | osa00750 | 4 | 8 | 2.9E-02 |
| Fructose and mannose metabolism | osa00051 | 13 | 58 | 3.0E-02 |
| Biosynthesis of unsaturated fatty acids | osa01040 | 9 | 34 | 3.0E-02 |
| Galactose metabolism | osa00052 | 12 | 53 | 3.4E-02 |
| Fatty acid elongation | osa00062 | 7 | 24 | 3.7E-02 |
| Sulfur relay system | osa04122 | 5 | 14 | 4.1E-02 |
| Fatty acid metabolism | osa01212 | 14 | 68 | 4.3E-02 |
| Folate biosynthesis | osa00790 | 6 | 20 | 4.7E-02 |
| Linoleic acid metabolism | osa00591 | 5 | 15 | 5.0E-02 |
| **KEGG analysis result for all DEGs at 3 day time point** | | | | |
| Ribosome | osa03010 | 122 | 337 | 4.6E-48 |
| DNA replication | osa03030 | 18 | 51 | 4.0E-08 |
| Ribosome biogenesis in eukaryotes | osa03008 | 21 | 86 | 6.0E-07 |
| Photosynthesis - antenna proteins | osa00196 | 8 | 15 | 2.5E-05 |
| Protein processing in endoplasmic reticulum | osa04141 | 25 | 189 | 5.6E-04 |
| Pyrimidine metabolism | osa00240 | 17 | 110 | 9.1E-04 |
| Mismatch repair | osa03430 | 9 | 40 | 1.6E-03 |
| RNA degradation | osa03018 | 15 | 97 | 1.8E-03 |
| Carotenoid biosynthesis | osa00906 | 7 | 27 | 2.7E-03 |
| Biosynthesis of secondary metabolites | osa01110 | 72 | 848 | 4.3E-03 |
| Purine metabolism | osa00230 | 17 | 130 | 4.4E-03 |
| Homologous recombination | osa03440 | 9 | 50 | 6.0E-03 |
| Phenylpropanoid biosynthesis | osa00940 | 16 | 125 | 6.8E-03 |
| Base excision repair | osa03410 | 8 | 42 | 7.0E-03 |
| Nucleotide excision repair | osa03420 | 10 | 66 | 1.1E-02 |
| Plant hormone signal transduction | osa04075 | 20 | 184 | 1.3E-02 |
| Linoleic acid metabolism | osa00591 | 4 | 15 | 2.1E-02 |
| alpha-Linolenic acid metabolism | osa00592 | 6 | 39 | 4.2E-02 |

**Supplementary Table S5. Clustering of time-series expression profile for DEGs.**

| **Pattern** | **Term** | **Category** | **Description** | **Number in input list** | **Number in ref** | **p-value** | **FDR** |
| --- | --- | --- | --- | --- | --- | --- | --- |
| Pattern 10 | GO:0050896 | Biological Process | response to stimulus | 101 | 5265 | 1.6E-05 | 4.5E-03 |
|  | GO:0003824 | Molecular Function | catalytic activity | 169 | 9920 | 4.9E-06 | 8.2E-05 |
|  | GO:0016772 | Molecular Function | transferase activity, transferring phosphorus-containing groups | 51 | 2020 | 4.8E-06 | 8.2E-05 |
|  | GO:0016301 | Molecular Function | kinase activity | 51 | 2020 | 4.8E-06 | 8.2E-05 |
|  | GO:0016740 | Molecular Function | transferase activity | 73 | 3913 | 8.1E-04 | 1.0E-02 |
|  | osa00480 | KEGG pathway | Glutathione metabolism | 6 | 73 | 4.1E-04 | 6.8E-03 |
|  | osa00940 | KEGG pathway | Phenylpropanoid biosynthesis | 7 | 125 | 1.2E-03 | 1.9E-02 |
| Pattern 9 | GO:0009607 | Biological Process | response to biotic stimulus | 43 | 1076 | 6.8E-12 | 1.6E-09 |
|  | GO:0050896 | Biological Process | response to stimulus | 105 | 5265 | 1.0E-08 | 1.2E-06 |
|  | GO:0006950 | Biological Process | response to stress | 73 | 3618 | 3.0E-06 | 2.4E-04 |
|  | GO:0009719 | Biological Process | response to endogenous stimulus | 38 | 1491 | 9.6E-06 | 5.7E-04 |
|  | GO:0008152 | Biological Process | metabolic process | 204 | 14183 | 1.4E-05 | 6.6E-04 |
|  | GO:0023052 | Biological Process | signaling | 40 | 1849 | 1.9E-04 | 7.6E-03 |
|  | GO:0007165 | Biological Process | signal transduction | 31 | 1464 | 1.5E-03 | 4.1E-02 |
|  | GO:0050794 | Biological Process | regulation of cellular process | 31 | 1464 | 1.5E-03 | 4.1E-02 |
|  | GO:0023046 | Biological Process | signaling process | 31 | 1481 | 1.7E-03 | 4.1E-02 |
|  | GO:0023060 | Biological Process | signal transmission | 31 | 1481 | 1.7E-03 | 4.1E-02 |
|  | GO:0006629 | Biological Process | lipid metabolic process | 23 | 991 | 2.0E-03 | 4.3E-02 |
|  | GO:0003824 | Molecular Function | catalytic activity | 155 | 9920 | 7.9E-06 | 4.6E-04 |
|  | osa00592 | KEGG pathway | alpha-Linolenic acid metabolism | 5 | 39 | 1.2E-04 | 2.3E-03 |
|  | osa00906 | KEGG pathway | Carotenoid biosynthesis | 4 | 27 | 3.5E-04 | 5.3E-03 |
| Pattern 6 | GO:0006259 | Biological Process | DNA metabolic process | 26 | 434 | 5.0E-13 | 1.3E-10 |
|  | GO:0006807 | Biological Process | nitrogen compound metabolic process | 65 | 3898 | 6.5E-06 | 5.6E-04 |
|  | GO:0006139 | Biological Process | nucleobase, nucleoside, nucleotide and nucleic acid metabolic process | 65 | 3898 | 6.5E-06 | 5.6E-04 |
|  | GO:0003677 | Molecular Function | DNA binding | 47 | 2434 | 4.7E-06 | 2.1E-04 |
|  | GO:0003676 | Molecular Function | nucleic acid binding | 54 | 3349 | 1.2E-04 | 2.8E-03 |
|  | osa03430 | KEGG pathway | Mismatch repair | 5 | 40 | 4.8E-05 | 5.5E-04 |
|  | osa03030 | KEGG pathway | DNA replication | 5 | 51 | 1.4E-04 | 1.3E-03 |
|  | osa03420 | KEGG pathway | Nucleotide excision repair | 5 | 66 | 4.2E-04 | 3.2E-03 |
|  | osa03440 | KEGG pathway | Homologous recombination | 4 | 50 | 1.3E-03 | 5.3E-03 |
|  | osa03410 | KEGG pathway | Base excision repair | 3 | 42 | 7.4E-03 | 2.4E-02 |
|  | osa03008 | KEGG pathway | Ribosome biogenesis in eukaryotes | 4 | 86 | 8.5E-03 | 2.6E-02 |
|  | osa00500 | KEGG pathway | Starch and sucrose metabolism | 5 | 159 | 1.6E-02 | 4.7E-02 |
| Pattern 5 | GO:0009791 | Biological Process | post-embryonic development | 26 | 1441 | 1.3E-04 | 1.8E-02 |
|  | GO:0007275 | Biological Process | multicellular organismal development | 38 | 2591 | 2.5E-04 | 1.8E-02 |
|  | GO:0032502 | Biological Process | developmental process | 40 | 2806 | 3.0E-04 | 1.8E-02 |
|  | GO:0032501 | Biological Process | multicellular organismal process | 38 | 2636 | 3.5E-04 | 1.8E-02 |
|  | GO:0000003 | Biological Process | reproduction | 24 | 1449 | 7.9E-04 | 3.3E-02 |
|  | osa00500 | KEGG pathway | Starch and sucrose metabolism | 5 | 159 | 8.6E-03 | 4.0E-02 |

**Supplementary Table S6. Allelopathy related genes identified previously in our DEGs.**

| **(a) Allelopathy genes identified previously in our DEGs at 3h time point.** | | | | | | | | | |
| --- | --- | --- | --- | --- | --- | --- | --- | --- | --- |
| **Sl. No.** | **Gene id** | **Locus** | **Putative Function** | **Previously identified Gene/Clone** | **Accession number** | **Reference** | **log2**  **(fold_change)** | **Up- or Down-regulated genes** | ***P* value** |
| 1 | LOC_Os01g01920 | Chr1:498453-506264 | HD domain containing protein 2, putative, expressed | *Clone 163* | CM000126 | Fang et al. (2009) | 0.49 | Up | 0.04 |
| 2 | LOC_Os01g02890 | Chr1:1046603-1053166 | phosphatidylserine synthase, putative, expressed | *Clone 163* | CM000126 | Fang et al. (2009) | 0.57 | Up | 0.02 |
| 3 | LOC_Os01g05610 | Chr1:2674399-2675373 | Core histone H2A/H2B/H3/H4 domain containing protein, putative, expressed | *DEG-5* | CT828153.1 | Junaedi et al. (2008) | 1.18 | Up | 0.00 |
| 4 | LOC_Os01g05900 | Chr1:2820035-2838580 | Core histone H2A/H2B/H3/H4 domain containing protein, putative, expressed | *DEG-5* | CT828153.1 | Junaedi et al. (2008) | -0.48 | Down | 0.01 |
| 5 | LOC_Os01g06010 | Chr1:2872109-2872988 | Core histone H2A/H2B/H3/H4 domain containing protein, putative, expressed | *DEG-5* | CT828153.1 | Junaedi et al. (2008) | -0.47 | Down | 0.01 |
| 6 | LOC_Os01g06270 | Chr1:2971124-2979313 | expressed protein | *Clone 163* | CM000126 | Fang et al. (2009) | 0.57 | Up | 0.00 |
| 7 | LOC_Os01g06600 | Chr1:3108116-3113963 | glutaryl-CoA dehydrogenase, mitochondrial precursor, putative, expressed | *Clone 163* | CM000126 | Fang et al. (2009) | 0.56 | Up | 0.00 |
| 8 | LOC_Os01g07870 | Chr1:3804527-3811030 | ABC transporter family protein, putative, expressed | *Clone 163* | CM000126 | Fang et al. (2009) | 0.81 | Up | 0.00 |
| 9 | LOC_Os01g09700 | Chr1:5002117-5005727 | aminotransferase, classes I and II, domain containing protein, expressed | *DEG-2* | AY522330.1 | Junaedi et al. (2008) | 1.93 | Up | 0.00 |
| 10 | LOC_Os01g10530 | Chr1:5591696-5599234 | expressed protein | *Clone 163* | CM000126 | Fang et al. (2009) | 0.51 | Up | 0.01 |
| 11 | LOC_Os01g14580 | Chr1:8170308-8177889 | dehydrogenase, putative, expressed | *Clone 163* | CM000126 | Fang et al. (2009) | 0.52 | Up | 0.02 |
| 12 | LOC_Os01g15020 | Chr1:8420922-8430280 | lissencephaly type-1-like homology motif, putative, expressed | *Clone 163* | CM000126 | Fang et al. (2009) | 0.40 | Up | 0.04 |
| 13 | LOC_Os01g16170 | Chr1:9133518-9140430 | PQ loop repeat domain containing protein, expressed | *Clone 163* | CM000126 | Fang et al. (2009) | 0.81 | Up | 0.00 |
| 14 | LOC_Os01g16260 | Chr1:9201353-9211788 | major facilitator superfamily antiporter, putative, expressed | *Clone 163* | CM000126 | Fang et al. (2009) | 0.85 | Up | 0.00 |
| 15 | LOC_Os01g17010 | Chr1:9738441-9744728 | phospholipid-transporting ATPase, putative, expressed | *Clone 163* | CM000126 | Fang et al. (2009) | 0.50 | Up | 0.01 |
| 16 | LOC_Os01g21034 | Chr1:11741376-11750709 | pectinesterase, putative, expressed | *Clone 163* | CM000126 | Fang et al. (2009) | -0.51 | Down | 0.00 |
| 17 | LOC_Os01g22249 | Chr1:12509591-12519255 | peroxidase precursor, putative, expressed | *Clone 265* | AC092263 | Fang et al. (2009) | 1.05 | Up | 0.03 |
| 18 | LOC_Os01g28680 | Chr1:16046648-16056268 | WD domain, G-beta repeat domain containing protein, expressed | *Clone 163* | CM000126 | Fang et al. (2009) | 0.75 | Up | 0.00 |
| 19 | LOC_Os01g31870 | Chr1:17454022-17465049 | natural resistance-associated macrophage protein, putative, expressed | *Clone 163* | CM000126 | Fang et al. (2009) | 0.97 | Up | 0.00 |
| 20 | LOC_Os01g34970 | Chr1:19352054-19359550 | MDR-like ABC transporter, putative, expressed | *Clone 163* | CM000126 | Fang et al. (2009) | 0.47 | Up | 0.04 |
| 21 | LOC_Os01g36080 | Chr1:19962456-19970111 | protein phosphatase 2C containing protein, expressed | *Clone 163* | CM000126 | Fang et al. (2009) | 0.57 | Up | 0.00 |
| 22 | LOC_Os01g38359 | Chr1:21525439-21549830 | peptidyl-prolyl cis-trans isomerase, FKBP-type, putative, expressed | *Clone 695* | AP003252 | Fang et al. (2009) | 1.00 | Up | 0.00 |
| 23 | LOC_Os01g40870 | Chr1:23113644-23119715 | aldehyde dehydrogenase, putative, expressed | *Clone 163* | CM000126 | Fang et al. (2009) | 1.25 | Up | 0.00 |
| 24 | LOC_Os01g42380 | Chr1:24075064-24082181 | pleiotropic drug resistance protein, putative, expressed | *Clone 163* | CM000126 | Fang et al. (2009) | 0.89 | Up | 0.00 |
| 25 | LOC_Os01g42410 | Chr1:24107079-24115143 | pleiotropic drug resistance protein, putative, expressed | *Clone 163* | CM000126 | Fang et al. (2009) | 1.16 | Up | 0.00 |
| 26 | LOC_Os01g42850 | Chr1:24380635-24386687 | ThiF family domain containing protein, putative, expressed | *Clone 163* | CM000126 | Fang et al. (2009) | 0.48 | Up | 0.01 |
| 27 | LOC_Os01g44220 | Chr1:25353768-25362199 | glucose-1-phosphate adenylyltransferase large subunit, chloroplast precursor, putative, expressed | *Clone 163* | CM000126 | Fang et al. (2009) | -1.15 | Down | 0.00 |
| 28 | LOC_Os01g44990 | Chr1:25524183-25530680 | ercc6 protein, putative, expressed | *Clone 163* | CM000126 | Fang et al. (2009) | 0.69 | Up | 0.01 |
| 29 | LOC_Os01g45274 | Chr1:25692716-25705090 | carbonic anhydrase, chloroplast precursor, putative, expressed | *Clone 163* | CM000126 | Fang et al. (2009) | 3.03 | Up | 0.00 |
| 30 | LOC_Os01g45880 | Chr1:26060787-26070899 | retrotransposon protein, putative, unclassified, expressed | *Clone 163* | CM000126 | Fang et al. (2009) | 0.76 | Up | 0.00 |
| 31 | LOC_Os01g45990 | Chr1:26119707-26126165 | potassium channel AKT1, putative, expressed | *Clone 163* | CM000126 | Fang et al. (2009) | 0.51 | Up | 0.01 |
| 32 | LOC_Os01g46750 | Chr1:26628888-26636949 | AMP-binding enzyme, putative, expressed | *Clone 163* | CM000126 | Fang et al. (2009) | 0.38 | Up | 0.04 |
| 33 | LOC_Os01g47460 | Chr1:27123485-27129401 | expressed protein | *Clone 163* | CM000126 | Fang et al. (2009) | 0.58 | Up | 0.02 |
| 34 | LOC_Os01g47530 | Chr1:27171286-27178431 | CGMC_MAPKCMGC_2.6 - CGMC includes CDA, MAPK, GSK3, and CLKC kinases, expressed | *Clone 163* | CM000126 | Fang et al. (2009) | 0.48 | Up | 0.01 |
| 35 | LOC_Os01g48390 | Chr1:27733408-27739844 | expressed protein | *Clone 163* | CM000126 | Fang et al. (2009) | 0.45 | Up | 0.02 |
| 36 | LOC_Os01g48790 | Chr1:27983687-27990383 | YT521-B-like family domain containing protein, expressed | *Clone 163* | CM000126 | Fang et al. (2009) | 0.40 | Up | 0.03 |
| 37 | LOC_Os01g49290 | Chr1:28330799-28333392 | WD repeat-containing protein, putative, expressed | *OsKSL4* | Os04g10060 | Xu et al. ( 2012) | -0.64 | Down | 0.01 |
| 38 | LOC_Os01g49614 | Chr1:28524005-28534608 | Protein kinase domain containing protein, expressed | *DEG-2* | AY522330.1 | Junaedi et al. (2008) | 0.74 | Up | 0.03 |
| 39 | LOC_Os01g50100 | Chr1:28786108-28793300 | ABC transporter, ATP-binding protein, putative, expressed | *Clone 163* | CM000126 | Fang et al. (2009) | 1.42 | Up | 0.00 |
| 40 | LOC_Os01g50160 | Chr1:28825744-28832656 | MDR-like ABC transporter, putative, expressed | *Clone 163* | CM000126 | Fang et al. (2009) | 0.95 | Up | 0.00 |
| 41 | LOC_Os01g51632 | Chr1:29662431-29667980 | myosin XI, putative, expressed | *OsKSL4* | Os04g10060 | Xu et al. ( 2012) | 0.72 | Up | 0.00 |
| 42 | LOC_Os01g52110 | Chr1:29965063-29970767 | RING finger and CHY zinc finger domain-containing protein 1, putative, expressed | *Clone 163* | CM000126 | Fang et al. (2009) | 0.63 | Up | 0.00 |
| 43 | LOC_Os01g52790 | Chr1:30372203-30376917 | cytochrome P450 72A1, putative, expressed | *OsKSL4* | Os04g10060 | Xu et al. ( 2012) | 0.99 | Up | 0.00 |
| 44 | LOC_Os01g53294 | Chr1:30622670-30634405 | respiratory burst oxidase protein B, putative, expressed | *Clone 163* | CM000126 | Fang et al. (2009) | 0.41 | Up | 0.03 |
| 45 | LOC_Os01g55360 | Chr1:31870931-31877607 | phosphatidate cytidylyltransferase, putative, expressed | *Clone 163* | CM000126 | Fang et al. (2009) | 0.42 | Up | 0.03 |
| 46 | LOC_Os01g55799 | Chr1:32137365-32143435 | exo70 exocyst complex subunit domain containing protein, expressed | *Clone 163* | CM000126 | Fang et al. (2009) | 0.48 | Up | 0.05 |
| 47 | LOC_Os01g56930 | Chr1:32886849-32892644 | eukaryotic aspartyl protease domain containing protein, expressed | *Clone 163* | CM000126 | Fang et al. (2009) | 0.67 | Up | 0.00 |
| 48 | LOC_Os01g56980 | Chr1:32910261-32925565 | histidine acid phosphatase, putative, expressed | *Clone 163* | CM000126 | Fang et al. (2009) | 0.56 | Up | 0.04 |
| 49 | LOC_Os01g57073 | Chr1:32965304-32977407 | insulin-degrading enzyme, putative, expressed | *Clone 163* | CM000126 | Fang et al. (2009) | 0.61 | Up | 0.00 |
| 50 | LOC_Os01g57210 | Chr1:33050634-33058497 | katanin p80 WD40 repeat-containing subunit B1 homolog 1, putative, expressed | *Clone 163* | CM000126 | Fang et al. (2009) | 0.47 | Up | 0.01 |
| 51 | LOC_Os01g57220 | Chr1:33059186-33067834 | secretory carrier-associated membrane protein, putative, expressed | *Clone 163* | CM000126 | Fang et al. (2009) | 0.45 | Up | 0.03 |
| 52 | LOC_Os01g58640 | Chr1:33897215-33904518 | nucleotide pyrophosphatase/phosphodiesterase, putative, expressed | *OsKSL4* | Os04g10060 | Xu et al. ( 2012) | -1.01 | Down | 0.00 |
| 53 | LOC_Os01g59570 | Chr1:34445742-34454439 | senescence-induced receptor-like serine/threonine-protein kinase precursor, putative, expressed | *OsKSL4* | Os04g10060 | Xu et al. ( 2012) | 1.43 | Up | 0.00 |
| 54 | LOC_Os01g60280 | Chr1:34868403-34879449 | ATP binding protein, putative, expressed | *Clone 163* | CM000126 | Fang et al. (2009) | 0.49 | Up | 0.01 |
| 55 | LOC_Os01g61170 | Chr1:35407929-35408598 | prenylated rab acceptor, putative, expressed | *Clone 512* | AP003734 | Fang et al. (2009) | -0.58 | Down | 0.02 |
| 56 | LOC_Os01g61190 | Chr1:35412929-35417584 | exo70 exocyst complex subunit, putative, expressed | *Clone 512* | AP003734 | Fang et al. (2009) | 0.89 | Up | 0.02 |
| 57 | LOC_Os01g61320 | Chr1:35477412-35479577 | thioredoxin, putative, expressed | *Clone 512* | AP003734 | Fang et al. (2009) | -0.44 | Down | 0.05 |
| 58 | LOC_Os01g61330 | Chr1:35480042-35482190 | ankyrin homolog precursor, putative, expressed | *Clone 512* | AP003734 | Fang et al. (2009) | -1.00 | Down | 0.00 |
| 59 | LOC_Os01g61350 | Chr1:35491909-35492921 | glutaredoxin, putative, expressed | *Clone 512* | AP003734 | Fang et al. (2009) | -0.97 | Down | 0.00 |
| 60 | LOC_Os01g61460 | Chr1:35538326-35544333 | leaf senescence related protein, putative, expressed | *Clone 695* | AP003252 | Fang et al. (2009) | 0.47 | Up | 0.01 |
| 61 | LOC_Os01g63220 | Chr1:36640942-36648018 | kinase, pfkB family, putative, expressed | *Clone 163* | CM000126 | Fang et al. (2009) | -0.51 | Down | 0.05 |
| 62 | LOC_Os01g64750 | Chr1:37566050-37575992 | sterol 3-beta-glucosyltransferase, putative, expressed | *Clone 163* | CM000126 | Fang et al. (2009) | 0.65 | Up | 0.01 |
| 63 | LOC_Os01g67330 | Chr1:39085080-39091029 | nucleotide-sugar transporter family protein, putative, expressed | *Clone 163* | CM000126 | Fang et al. (2009) | 0.43 | Up | 0.03 |
| 64 | LOC_Os01g67580 | Chr1:39275856-39282884 | multidrug resistance-associated protein, putative, expressed | *Clone 163* | CM000126 | Fang et al. (2009) | 0.63 | Up | 0.00 |
| 65 | LOC_Os01g68260 | Chr1:39666654-39670332 | lysine ketoglutarate reductase trans-splicing related 1, putative, expressed | *OsKSL4* | Os04g10060 | Xu et al. ( 2012) | 1.15 | Up | 0.04 |
| 66 | LOC_Os01g68680 | Chr1:39881006-39890797 | tetratricopeptide repeat domain containing protein, expressed | *Clone 163* | CM000126 | Fang et al. (2009) | 0.46 | Up | 0.03 |
| 67 | LOC_Os01g69920 | Chr1:40403714-40409615 | histidine kinase, putative, expressed | *Clone 163* | CM000126 | Fang et al. (2009) | 0.50 | Up | 0.01 |
| 68 | LOC_Os01g72430 | Chr1:42003930-42009785 | NADPH quinone oxidoreductase, putative, expressed | *Clone 163* | CM000126 | Fang et al. (2009) | 0.37 | Up | 0.05 |
| 69 | LOC_Os01g72990 | Chr1:42336216-42349399 | expressed protein | *Clone 163* | CM000126 | Fang et al. (2009) | 0.88 | Up | 0.00 |
| 70 | LOC_Os01g73170 | Chr1:42429501-42432618 | peroxidase precursor, putative, expressed | *Clone 593* | AP003263 | Fang et al. (2009) | -0.61 | Down | 0.01 |
| 71 | LOC_Os01g73200 | Chr1:42441659-42442958 | peroxidase precursor, putative, expressed | *Clone 593* | AP003263 | Fang et al. (2009) | 1.11 | Up | 0.00 |
| 72 | LOC_Os01g73250 | Chr1:42460554-42462334 | abscisic stress-ripening, putative, expressed | *Clone 593* | AP003263 | Fang et al. (2009) | -1.17 | Down | 0.00 |
| 73 | LOC_Os01g73270 | Chr1:42470326-42471850 | expressed protein | *Clone 593* | AP003263 | Fang et al. (2009) | -2.23 | Down | 0.00 |
| 74 | LOC_Os02g06630 | Chr2:3335546-3339094 | peroxidase precursor, putative, expressed | *OsCPS4* | Os04g09900 | Xu et al. ( 2012) | -1.10 | Down | 0.02 |
| 75 | LOC_Os02g15740 | Chr2:8869107-8871520 | expressed protein | *OsKSL4* | Os04g10060 | Xu et al. ( 2012) | -1.48 | Down | 0.00 |
| 76 | LOC_Os02g17680 | Chr2:10181425-10189201 | ethylene-responsive protein related, putative, expressed | *OsKSL4* | Os04g10060 | Xu et al. ( 2012) | 0.50 | Up | 0.01 |
| 77 | LOC_Os02g20360 | Chr2:11997093-12002633 | tyrosine aminotransferase, putative, expressed | DEG-6 | AK243448.1 | Junaedi et al. (2008) | 0.64 | Up | 0.00 |
| 78 | LOC_Os02g20490 | Chr2:12086016-12089352 | expressed protein | *Clone 512* | AP003734 | Fang et al. (2009) | -0.47 | Down | 0.03 |
| 79 | LOC_Os02g25580 | Chr2:14942532-14962091 | Sec1 family transport protein, putative, expressed | *DEG-2* | AY522330.1 | Junaedi et al. (2008) | 0.59 | Up | 0.00 |
| 80 | LOC_Os02g26600 | Chr2:15614927-15619328 | ATP binding, related, putative, expressed | *Clone 512* | AP003734 | Fang et al. (2009) | -1.07 | Down | 0.00 |
| 81 | LOC_Os02g29510 | Chr2:17527549-17539534 | DUF803 domain containing, putative, expressed | *OsKSL4* | Os04g10060 | Xu et al. ( 2012) | 0.85 | Up | 0.03 |
| 82 | LOC_Os02g32690 | Chr2:19405390-19420793 | pleiotropic drug resistance protein 15, putative, expressed | *DEG-2* | AY522330.1 | Junaedi et al. (2008) | 0.83 | Up | 0.00 |
| 83 | LOC_Os02g36140 | Chr2:21765008-21773418 | terpene synthase, putative, expressed | *OsKSL4* | Os04g10060 | Xu et al. ( 2012) | 0.57 | Up | 0.02 |
| 84 | LOC_Os02g36340 | Chr2:21930616-21935565 | riboflavin biosynthesis protein ribAB, chloroplast precursor, putative, expressed | *Clone 512* | AP003734 | Fang et al. (2009) | 0.43 | Up | 0.02 |
| 85 | LOC_Os02g37654 | Chr2:22722658-22733252 | lecithin:cholesterol acyltransferase, putative, expressed | *DEG-7* | AC122144.1 | Junaedi et al. (2008) | 2.76 | Up | 0.00 |
| 86 | LOC_Os02g41460 | Chr2:24830540-24832108 | DUF640 domain containing protein, putative, expressed | *Clone 512* | AP003734 | Fang et al. (2009) | -0.96 | Down | 0.00 |
| 87 | LOC_Os02g56610 | Chr2:34687623-34689723 | DUF640 domain containing protein, putative, expressed | *Clone 512* | AP003734 | Fang et al. (2009) | -1.63 | Down | 0.00 |
| 88 | LOC_Os03g03034 | Chr3:1235372-1245060 | flavonol synthase/flavanone 3-hydroxylase, putative, expressed | *Clone 265* | AC092263 | Fang et al. (2009) | 1.13 | Up | 0.00 |
| 89 | LOC_Os03g04570 | Chr3:2131971-2136725 | peptide transporter PTR3-A, putative, expressed | *OsKSL4* | Os04g10060 | Xu et al. ( 2012) | 0.97 | Up | 0.00 |
| 90 | LOC_Os03g05330 | Chr3:2593128-2605502 | HEAT repeat family protein, putative, expressed | *DEG-2* | AY522330.1 | Junaedi et al. (2008) | 0.54 | Up | 0.00 |
| 91 | LOC_Os03g05530 | Chr3:2747955-2753329 | nodulin, putative, expressed | *Clone 512* | AP003734 | Fang et al. (2009) | 0.77 | Up | 0.00 |
| 92 | LOC_Os03g10478 | Chr3:5331109-5335911 | glycosyl hydrolase family 10 protein, putative, expressed | *Clone 265* | AC092263 | Fang et al. (2009) | -1.96 | Down | 0.00 |
| 93 | LOC_Os03g19480 | Chr3:10952132-10962384 | SET domain containing protein, expressed | *Clone 512* | AP003734 | Fang et al. (2009) | 0.46 | Up | 0.05 |
| 94 | LOC_Os03g22670 | Chr3:13087396-13093802 | triacylglycerol Lipase, putative, expressed | *OsKSL4* | Os04g10060 | Xu et al. ( 2012) | 0.56 | Up | 0.05 |
| 95 | LOC_Os03g26460 | Chr3:15118427-15121529 | CS domain containing protein, putative, expressed | *Clone 743 (V)* | NM_001056776 | Song et al. (2008) | 0.42 | Up | 0.04 |
| 96 | LOC_Os03g36750 | Chr3:20379615-20387075 | cbbY, putative, expressed | *DEG-7* | AC122144.1 | Junaedi et al. (2008) | 0.66 | Up | 0.00 |
| 97 | LOC_Os03g43750 | Chr3:24466602-24473610 | retrotransposon, putative, centromere-specific, expressed | *DEG-7* | AC122144.1 | Junaedi et al. (2008) | 1.22 | Up | 0.01 |
| 98 | LOC_Os03g45710 | Chr3:25800414-25801331 | 2Fe-2S iron-sulfur cluster binding domain containing protein, expressed | *DEG-2* | AY522330.1 | Junaedi et al. (2008) | -0.57 | Down | 0.02 |
| 99 | LOC_Os03g46190 | Chr3:26114965-26119703 | parafibromin, putative, expressed | *Clone 512* | AP003734 | Fang et al. (2009) | -0.53 | Down | 0.02 |
| 100 | LOC_Os03g47730 | Chr3:27050992-27053998 | homeobox domain containing protein, expressed | *OsKSL4* | Os04g10060 | Xu et al. ( 2012) | -0.65 | Down | 0.03 |
| 101 | LOC_Os03g57690 | Chr3:32879565-32886266 | aldehyde oxidase, putative, expressed | *OsKSL4* | Os04g10060 | Xu et al. ( 2012) | 0.41 | Up | 0.02 |
| 102 | LOC_Os03g64219 | Chr3:36286351-36289215 | OTU-like cysteine protease family protein, putative, expressed | *Clone 265* | AC092263 | Fang et al. (2009) | 0.54 | Up | 0.04 |
| 103 | LOC_Os03g64250 | Chr3:36295245-36299447 | expressed protein | *Clone 265* | AC092263 | Fang et al. (2009) | 0.55 | Up | 0.02 |
| 104 | LOC_Os03g64340 | Chr3:36366367-36367770 | heavy metal-associated domain containing protein, expressed | *Clone 265* | AC092263 | Fang et al. (2009) | 0.70 | Up | 0.01 |
| 105 | LOC_Os04g02640 | Chr4:997303-999140 | 3-ketoacyl-CoA synthase 6, putative, expressed | *OsKSL4* | Os04g10060 | Xu et al. ( 2012) | -1.16 | Down | 0.00 |
| 106 | LOC_Os04g02754 | Chr4:1054608-1070954 | amidase family protein, putative, expressed | *Clone 163* | CM000126 | Fang et al. (2009) | 0.61 | Up | 0.01 |
| 107 | LOC_Os04g09900 | Chr4:5318059-5326427 | ent-kaurene synthase, chloroplast precursor, putative, expressed | *OsCPS4* | Os04g09900 | Xu et al. ( 2012) | 1.27 | Up | 0.00 |
| 108 | LOC_Os04g16740 | Chr4:9123601-9125252 | ATP synthase subunit alpha, putative, expressed | *DEG-2* | AY522330.1 | Junaedi et al. (2008) | 0.46 | Up | 0.04 |
| 109 | LOC_Os04g38450 | Chr4:22855358-22861007 | gamma-glutamyltranspeptidase 1 precursor, putative, expressed | *OsKSL4* | Os04g10060 | Xu et al. ( 2012) | 0.72 | Up | 0.00 |
| 110 | LOC_Os04g42470 | Chr4:25126714-25134362 | regulatory subunit, putative, expressed | *OsKSL4* | Os04g10060 | Xu et al. ( 2012) | 0.76 | Up | 0.00 |
| 111 | LOC_Os04g43050 | Chr4:25474092-25489003 | Dicer, putative, expressed | *OsKSL4* | Os04g10060 | Xu et al. ( 2012) | 0.40 | Up | 0.04 |
| 112 | LOC_Os04g43580 | Chr4:25790013-25791567 | DUF640 domain containing protein, putative, expressed | *Clone 512* | AP003734 | Fang et al. (2009) | -1.98 | Down | 0.00 |
| 113 | LOC_Os04g43916 | Chr4:26023935-26035492 | expressed protein | *OsKSL4* | Os04g10060 | Xu et al. ( 2012) | 0.47 | Up | 0.01 |
| 114 | LOC_Os04g46450 | Chr4:27551357-27560074 | zinc finger, C3HC4 type domain containing protein, expressed | *OsKSL4* | Os04g10060 | Xu et al. ( 2012) | 0.37 | Up | 0.04 |
| 115 | LOC_Os04g49194 | Chr4:29331562-29338265 | naringenin,2-oxoglutarate 3-dioxygenase, putative, expressed | *OsKSL4* | Os04g10060 | Xu et al. ( 2012) | 1.47 | Up | 0.00 |
| 116 | LOC_Os04g54474 | Chr4:32403335-32412065 | transcription factor, putative, expressed | *OsKSL4* | Os04g10060 | Xu et al. ( 2012) | 0.47 | Up | 0.01 |
| 117 | LOC_Os04g56400 | Chr4:33624331-33631095 | glutamine synthetase, catalytic domain containing protein, expressed | *Clone 512* | AP003734 | Fang et al. (2009) | 0.91 | Up | 0.00 |
| 118 | LOC_Os04g58070 | Chr4:34586365-34588232 | aspartic proteinase nepenthesin precursor, putative, expressed | *OsKSL4* | Os04g10060 | Xu et al. ( 2012) | 1.12 | Up | 0.00 |
| 119 | LOC_Os05g04890 | Chr5:2351572-2354876 | expressed protein | *OsKSL4* | Os04g10060 | Xu et al. ( 2012) | -0.55 | Down | 0.01 |
| 120 | LOC_Os05g06980 | Chr5:3652661-3663546 | expressed protein | *OsCPS4* | Os04g09900 | Xu et al. ( 2012) | 0.38 | Up | 0.04 |
| 121 | LOC_Os05g16824 | Chr5:9585979-9595470 | SHR5-receptor-like kinase, putative, expressed | *Clone 512* | AP003734 | Fang et al. (2009) | 0.62 | Up | 0.00 |
| 122 | LOC_Os05g33110 | Chr5:19415388-19423248 | endo-1,3;1,4-beta-D-glucanase precursor, putative, expressed | *OsKSL4* | Os04g10060 | Xu et al. ( 2012) | 1.26 | Up | 0.00 |
| 123 | LOC_Os05g36270 | Chr5:21493838-21496838 | fructose-1,6-bisphosphatase, putative, expressed | *OsKSL4* | Os04g10060 | Xu et al. ( 2012) | -0.79 | Down | 0.00 |
| 124 | LOC_Os05g38730 | Chr5:22725526-22734013 | SAC3/GANP family protein, putative, expressed | *OsKSL4* | Os04g10060 | Xu et al. ( 2012) | 0.54 | Up | 0.01 |
| 125 | LOC_Os05g39450 | Chr5:23138672-23139858 | glutaredoxin, putative, expressed | *Clone 512* | AP003734 | Fang et al. (2009) | -1.04 | Down | 0.01 |
| 126 | LOC_Os05g39500 | Chr5:23178204-23178921 | DUF640 domain containing protein, putative, expressed | *Clone 512* | AP003734 | Fang et al. (2009) | -1.36 | Down | 0.01 |
| 127 | LOC_Os05g39670 | Chr5:23284632-23285757 | prenylated rab acceptor, putative, expressed | *Clone 512* | AP003734 | Fang et al. (2009) | -0.84 | Down | 0.00 |
| 128 | LOC_Os05g41180 | Chr5:24122437-24126677 | peptidase, T1 family, putative, expressed | *OsKSL4* | Os04g10060 | Xu et al. ( 2012) | 0.37 | Up | 0.04 |
| 129 | LOC_Os05g42300 | Chr5:24738574-24742551 | NAF1 domain containing protein, expressed | *OsKSL4* | Os04g10060 | Xu et al. ( 2012) | -0.44 | Down | 0.03 |
| 130 | LOC_Os05g46030 | Chr5:26685978-26704758 | myosin head family protein, expressed | *Clone 265* | AC092263 | Fang et al. (2009) | 0.46 | Up | 0.05 |
| 131 | LOC_Os05g47670 | Chr5:27317412-27320555 | zinc finger, C3HC4 type domain containing protein, expressed | *OsKSL4* | Os04g10060 | Xu et al. ( 2012) | 0.76 | Up | 0.00 |
| 132 | LOC_Os05g51110 | Chr5:29317523-29324891 | expressed protein | *OsKSL4* | Os04g10060 | Xu et al. ( 2012) | 0.78 | Up | 0.00 |
| 133 | LOC_Os06g01934 | Chr6:523052-530057 | homeobox domain containing protein, expressed | *Clone 265* | AC092263 | Fang et al. (2009) | 1.31 | Up | 0.00 |
| 134 | LOC_Os06g01950 | Chr6:530533-532108 | phosphoglycerate mutase, putative, expressed | *OsKSL4* | Os04g10060 | Xu et al. ( 2012) | 0.93 | Up | 0.02 |
| 135 | LOC_Os06g05630 | Chr6:2546687-2549257 | GDSL-like lipase/acylhydrolase, putative, expressed | *OsKSL4* | Os04g10060 | Xu et al. ( 2012) | 0.81 | Up | 0.00 |
| 136 | LOC_Os06g19590 | Chr6:11170410-11174891 | estradiol 17-beta-dehydrogenase 12, putative, expressed | *Clone 695* | AP003252 | Fang et al. (2009) | -0.59 | Down | 0.05 |
| 137 | LOC_Os06g19990 | Chr6:11461522-11464902 | GPI-anchored protein, putative, expressed | *OsKSL4* | Os04g10060 | Xu et al. ( 2012) | 0.49 | Up | 0.03 |
| 138 | LOC_Os06g27680 | Chr6:15665966-15670539 | retrotransposon protein, putative, Ty1-copia subclass, expressed | *OsKSL4* | Os04g10060 | Xu et al. ( 2012) | -0.65 | Down | 0.04 |
| 139 | LOC_Os06g29260 | Chr6:16697272-16717477 | retrotransposon protein, putative, unclassified, expressed | *Clone 587* | AP005578 | Fang et al. (2009) | 1.86 | Up | 0.02 |
| 140 | LOC_Os06g36560 | Chr6:21478407-21482740 | inositol oxygenase, putative, expressed | *Clone 512* | AP003734 | Fang et al. (2009) | 1.02 | Up | 0.00 |
| 141 | LOC_Os06g37300 | Chr6:22020839-22028146 | cytochrome P450, putative, expressed | *OsCPS4* | Os04g09900 | Xu et al. ( 2012) | 0.85 | Up | 0.01 |
| 142 | LOC_Os06g39752 | Chr6:23586380-23586782 | expressed protein | *DEG-2* | AY522330.1 | Junaedi et al. (2008) | 0.00 | Down | 0.00 |
| 143 | LOC_Os06g39875 | Chr6:23689552-23691740 | ras-related protein, putative, expressed | *OsKSL4* | Os04g10060 | Xu et al. ( 2012) | -1.37 | Down | 0.04 |
| 144 | LOC_Os06g43870 | Chr6:26421511-26427830 | expressed protein | *DEG-2* | AY522330.1 | Junaedi et al. (2008) | 0.54 | Up | 0.01 |
| 145 | LOC_Os06g44620 | Chr6:26943093-26949889 | AMP-binding domain containing protein, expressed | *Clone 512* | AP003734 | Fang et al. (2009) | 0.43 | Up | 0.02 |
| 146 | LOC_Os06g46149 | Chr6:27960868-27971323 | serine/arginine repetitive matrix protein 1, putative, expressed | *Clone 512* | AP003734 | Fang et al. (2009) | 1.35 | Up | 0.00 |
| 147 | LOC_Os06g46284 | Chr6:28045146-28058082 | glycosyl hydrolase, family 31, putative, expressed | *DEG-7* | AC122144.1 | Junaedi et al. (2008) | -0.76 | Down | 0.00 |
| 148 | LOC_Os06g47850 | Chr6:28958625-28960418 | zinc finger family protein, putative, expressed | *OsKSL4* | Os04g10060 | Xu et al. ( 2012) | 1.60 | Up | 0.00 |
| 149 | LOC_Os07g13634 | Chr7:7815441-7832311 | cytokinin-N-glucosyltransferase 1, putative, expressed | *Clone 163* | CM000126 | Fang et al. (2009) | -1.16 | Down | 0.03 |
| 150 | LOC_Os07g19530 | Chr7:11551088-11570302 | peptidase, putative, expressed | *Clone 695* | AP003252 | Fang et al. (2009) | 0.61 | Up | 0.00 |
| 151 | LOC_Os07g32420 | Chr7:19281737-19289773 | bromodomain domain containing protein, expressed | *DEG-2* | AY522330.1 | Junaedi et al. (2008) | 0.43 | Up | 0.02 |
| 152 | LOC_Os07g38280 | Chr7:22975173-22991258 | insulin-degrading enzyme, putative, expressed | *DEG-2* | AY522330.1 | Junaedi et al. (2008) | 0.56 | Up | 0.03 |
| 153 | LOC_Os07g40550 | Chr7:24292559-24298863 | IBS1, putative, expressed | *OsKSL4* | Os04g10060 | Xu et al. ( 2012) | 0.44 | Up | 0.01 |
| 154 | LOC_Os07g40940 | Chr7:24505165-24508963 | X8 domain containing protein, expressed | *OsKSL4* | Os04g10060 | Xu et al. ( 2012) | -0.37 | Down | 0.05 |
| 155 | LOC_Os07g43925 | Chr7:26256477-26259188 | expressed protein | *Clone 695* | AP003252 | Fang et al. (2009) | -1.10 | Down | 0.00 |
| 156 | LOC_Os07g48330 | Chr7:28882153-28886452 | cytochrome P450, putative, expressed | *Clone 512* | AP003734 | Fang et al. (2009) | -2.41 | Down | 0.00 |
| 157 | LOC_Os08g05620 | Chr8:3007240-3009195 | cytochrome P450, putative, expressed | *OsKSL4* | Os04g10060 | Xu et al. ( 2012) | -0.60 | Down | 0.02 |
| 158 | LOC_Os08g14195 | Chr8:8479360-8481824 | expressed protein | *OsCPS4* | Os04g09900 | Xu et al. ( 2012) | 0.45 | Up | 0.02 |
| 159 | LOC_Os08g20544 | Chr8:12331525-12342183 | expressed protein | *Clone 512* | AP003734 | Fang et al. (2009) | 0.54 | Up | 0.01 |
| 160 | LOC_Os08g24946 | Chr8:15115419-15130251 | EDM2, putative, expressed | *DEG-2* | AY522330.1 | Junaedi et al. (2008) | 0.43 | Up | 0.02 |
| 161 | LOC_Os08g29124 | Chr8:17822366-17838135 | expressed protein | *DEG-2* | AY522330.1 | Junaedi et al. (2008) | 0.47 | Up | 0.02 |
| 162 | LOC_Os08g29590 | Chr8:18156706-18160981 | zinc finger, C3HC4 type family protein, expressed | *DEG-2* | AY522330.1 | Junaedi et al. (2008) | 0.49 | Up | 0.03 |
| 163 | LOC_Os08g29770 | Chr8:18305363-18312142 | endoglucanase, putative, expressed | *Clone 265* | AC092263 | Fang et al. (2009) | -1.45 | Down | 0.02 |
| 164 | LOC_Os08g32850 | Chr8:20359561-20366443 | methylcrotonoyl-CoA carboxylase beta chain, mitochondrial precursor, putative, expressed | *Clone 512* | AP003734 | Fang et al. (2009) | 0.50 | Up | 0.01 |
| 165 | LOC_Os08g33650 | Chr8:21014457-21018732 | SET domain-containing protein, putative, expressed | *DEG-2* | AY522330.1 | Junaedi et al. (2008) | -0.68 | Down | 0.00 |
| 166 | LOC_Os08g39140 | Chr8:24719085-24723553 | heat shock protein, putative, expressed | *OsKSL4* | Os04g10060 | Xu et al. ( 2012) | 0.58 | Up | 0.02 |
| 167 | LOC_Os08g44220 | Chr8:27834546-27838106 | GLTP domain containing protein, putative, expressed | *Clone 512* | AP003734 | Fang et al. (2009) | 0.88 | Up | 0.02 |
| 168 | LOC_Os08g44850 | Chr8:28170357-28172330 | C2 domain containing protein, putative, expressed | *OsKSL4* | Os04g10060 | Xu et al. ( 2012) | -0.73 | Down | 0.00 |
| 169 | LOC_Os09g04730 | Chr9:2512440-2517277 | dehydrogenase/reductase SDR family member 2, putative, expressed | *Clone 512* | AP003734 | Fang et al. (2009) | 0.59 | Up | 0.00 |
| 170 | LOC_Os09g07320 | Chr9:3620062-3633216 | ethanol tolerance protein GEKO1, putative, expressed | *Clone 512* | AP003734 | Fang et al. (2009) | 0.51 | Up | 0.04 |
| 171 | LOC_Os09g10054 | Chr9:5471799-5491310 | disease resistance protein RPS2, putative, expressed | *Clone 265* | AC092263 | Fang et al. (2009) | 0.85 | Up | 0.02 |
| 172 | LOC_Os09g19954 | Chr9:11946746-11952503 | ribosomal protein, putative, expressed | *DEG-2* | AY522330.1 | Junaedi et al. (2008) | 0.73 | Up | 0.04 |
| 173 | LOC_Os09g25490 | Chr9:15281267-15285910 | CESA9 - cellulose synthase, expressed | *Clone 670* | AF200528 | Fang et al. (2009) | 0.58 | Up | 0.00 |
| 174 | LOC_Os09g36700 | Chr9:21165181-21167650 | ribonuclease T2 family domain containing protein, expressed | *OsKSL4* | Os04g10060 | Xu et al. ( 2012) | -0.98 | Down | 0.00 |
| 175 | LOC_Os10g04860 | Chr10:2358789-2368732 | aldehyde oxidase, putative, expressed | *OsCPS4* | Os04g09900 | Xu et al. ( 2012) | 0.51 | Up | 0.02 |
| 176 | LOC_Os10g21268 | Chr10:10861157-10862712 | ribulose bisphosphate carboxylase large chain precursor, putative, expressed | *DEG-2* | AY522330.1 | Junaedi et al. (2008) | 0.77 | Up | 0.00 |
| 177 | LOC_Os10g25180 | Chr10:12997652-13001193 | phosphoinositide phosphatase family protein, putative, expressed | *OsKSL4* | Os04g10060 | Xu et al. ( 2012) | -1.94 | Down | 0.00 |
| 178 | LOC_Os10g25550 | Chr10:13219949-13226158 | tyrosine protein kinase domain containing protein, putative, expressed | *DEG-7* | AC122144.1 | Junaedi et al. (2008) | 0.61 | Up | 0.03 |
| 179 | LOC_Os10g25674 | Chr10:13289611-13292227 | mps one binder kinase activator-like 1A, putative, expressed | *DEG-7* | AC122144.1 | Junaedi et al. (2008) | 0.68 | Up | 0.01 |
| 180 | LOC_Os10g32980 | Chr10:17261951-17266613 | CESA7 - cellulose synthase, expressed | *Clone 670* | AF200528 | Fang et al. (2009) | 0.45 | Up | 0.02 |
| 181 | LOC_Os10g33650 | Chr10:17739201-17744567 | CK1_CaseinKinase_1.9 - CK1 includes the casein kinase 1 kinases, expressed | *Clone 512* | AP003734 | Fang et al. (2009) | 0.59 | Up | 0.05 |
| 182 | LOC_Os10g33780 | Chr10:17888296-17889724 | DUF640 domain containing protein, putative, expressed | *Clone 512* | AP003734 | Fang et al. (2009) | -0.90 | Down | 0.00 |
| 183 | LOC_Os10g39140 | Chr10:20886351-20892217 | flavonol synthase/flavanone 3-hydroxylase, putative, expressed | *Clone 265* | AC092263 | Fang et al. (2009) | 0.47 | Up | 0.01 |
| 184 | LOC_Os10g39680 | Chr10:21205699-21207611 | CHIT14 - Chitinase family protein precursor, expressed | *OsCPS4* | Os04g09900 | Xu et al. ( 2012) | 1.68 | Up | 0.00 |
| 185 | LOC_Os10g41970 | Chr10:22568901-22573486 | methyltransferase, putative, expressed | *OsKSL4* | Os04g10060 | Xu et al. ( 2012) | 0.54 | Up | 0.03 |
| 186 | LOC_Os11g02200 | Chr11:618436-620123 | expressed protein | *DEG-2* | AY522330.1 | Junaedi et al. (2008) | 0.74 | Up | 0.05 |
| 187 | LOC_Os11g05480 | Chr11:2462253-2468733 | transcription factor, putative, expressed | *Clone 512* | AP003734 | Fang et al. (2009) | 1.23 | Up | 0.00 |
| 188 | LOC_Os11g35090 | Chr11:20571983-20577400 | kinesin motor domain containing protein, putative, expressed | *Clone 512* | AP003734 | Fang et al. (2009) | -0.49 | Down | 0.02 |
| 189 | LOC_Os11g37740 | Chr11:22310965-22319156 | stripe rust resistance protein Yr10, putative, expressed | *OsKSL4* | Os04g10060 | Xu et al. ( 2012) | 0.60 | Up | 0.01 |
| 190 | LOC_Os11g47809 | Chr11:28827675-28828513 | metallothionein, putative, expressed | *Clone 715 (IV)* | NM_001075076 | Song et al. (2008) | 1.01 | Up | 0.00 |
| 191 | LOC_Os12g02470 | Chr12:836515-838755 | WRKY65, expressed | *Clone 695* | AP003252 | Fang et al. (2009) | 3.11 | Up | 0.00 |
| 192 | LOC_Os12g10580 | Chr12:5614139-5618055 | ribulose bisphosphate carboxylase large chain precursor, putative, expressed | *DEG-2* | AY522330.1 | Junaedi et al. (2008) | 0.90 | Up | 0.01 |
| 193 | LOC_Os12g13270 | Chr12:7374995-7384594 | expressed protein | *Clone 512* | AP003734 | Fang et al. (2009) | 0.67 | Up | 0.00 |
| 194 | LOC_Os12g19381 | Chr12:11262562-11278448 | ribulose bisphosphate carboxylase small chain, chloroplast precursor, putative, expressed | *Clone 695* | AP003252 | Fang et al. (2009) | 0.89 | Up | 0.00 |
| 195 | LOC_Os12g24650 | Chr12:14109287-14114508 | leucine aminopeptidase, chloroplast precursor, putative, expressed | *OsKSL4* | Os04g10060 | Xu et al. ( 2012) | 2.17 | Up | 0.00 |
| 196 | LOC_Os12g26290 | Chr12:15330145-15340505 | alpha-DOX2, putative, expressed | *Clone 512* | AP003734 | Fang et al. (2009) | 1.29 | Up | 0.00 |
| 197 | LOC_Os12g31460 | Chr12:18918229-18920690 | heat shock protein DnaJ, putative, expressed | *OsKSL4* | Os04g10060 | Xu et al. ( 2012) | 0.64 | Up | 0.03 |
| 198 | LOC_Os12g31860 | Chr12:19180633-19185917 | ureide permease, putative, expressed | *OsKSL4* | Os04g10060 | Xu et al. ( 2012) | 1.53 | Up | 0.00 |
| 199 | LOC_Os12g34010 | Chr12:20531997-20533727 | expressed protein | *DEG-2* | AY522330.1 | Junaedi et al. (2008) | -1.03 | Down | 0.02 |
| 200 | LOC_Os12g37510 | Chr12:23015818-23017657 | UDP-glucoronosyl and UDP-glucosyl transferase domain containing protein, expressed | *OsKSL4* | Os04g10060 | Xu et al. ( 2012) | -1.02 | Down | 0.00 |
| 201 | LOC_Os12g38051 | Chr12:23382983-23391427 | metallothionein, putative, expressed | *Clone 265* | AC092263 | Fang et al. (2009) | 1.97 | Up | 0.00 |
| **(b) Allelopathy genes identified previously in our DEGs at 3d time point.** | | | | | | | | |  |
| **Sl. No.** | **Gene id** | **Locus** | **Putative Function** | **Previously identified Gene/Clone** | **Accession number** | **Reference** | **log2**  **(fold_change)** | **Up- or Down-regulated genes** | ***P* value** |
| 1 | LOC_Os01g02890 | Chr1:1046603-1053166 | phosphatidylserine synthase, putative, expressed | *Clone 163* | CM000126 | Fang et al. (2009) | -0.92 | Down | 0.00 |
| 2 | LOC_Os01g03070 | Chr1:1171808-1179372 | transposon protein, putative, unclassified, expressed | *Clone 163* | CM000126 | Fang et al. (2009) | 0.46 | Up | 0.04 |
| 3 | LOC_Os01g05610 | Chr1:2674399-2675373 | Core histone H2A/H2B/H3/H4 domain containing protein, putative, expressed | *DEG-5* | CT828153.1 | Junaedi et al. (2008) | 0.78 | Up | 0.01 |
| 4 | LOC_Os01g05630 | Chr1:2686728-2687700 | Core histone H2A/H2B/H3/H4 domain containing protein, putative, expressed | *DEG-5* | CT828153.1 | Junaedi et al. (2008) | 0.73 | Up | 0.00 |
| 5 | LOC_Os01g05900 | Chr1:2820035-2838580 | Core histone H2A/H2B/H3/H4 domain containing protein, putative, expressed | *DEG-5* | CT828153.1 | Junaedi et al. (2008) | 0.53 | Up | 0.03 |
| 6 | LOC_Os01g05970 | Chr1:2844428-2858134 | OsFBO1 - F-box and other domain containing protein, expressed | *DEG-5* | CT828153.1 | Junaedi et al. (2008) | 0.78 | Up | 0.00 |
| 7 | LOC_Os01g06010 | Chr1:2872109-2872988 | Core histone H2A/H2B/H3/H4 domain containing protein, putative, expressed | *DEG-5* | CT828153.1 | Junaedi et al. (2008) | 0.52 | Up | 0.02 |
| 8 | LOC_Os01g12890 | Chr1:7157779-7164385 | expressed protein | *Clone 163* | CM000126 | Fang et al. (2009) | 0.53 | Up | 0.05 |
| 9 | LOC_Os01g31360 | Chr1:17151654-17157856 | expressed protein | *Clone 163* | CM000126 | Fang et al. (2009) | 0.49 | Up | 0.03 |
| 10 | LOC_Os01g33040 | Chr1:18150893-18157844 | kinesin motor domain containing protein, expressed | *Clone 163* | CM000126 | Fang et al. (2009) | 0.78 | Up | 0.00 |
| 11 | LOC_Os01g36240 | Chr1:20061960-20066530 | peroxidase precursor, putative, expressed | *Clone 265* | AC092263 | Fang et al. (2009) | 0.71 | Up | 0.02 |
| 12 | LOC_Os01g40980 | Chr1:23174816-23186976 | helicase, putative, expressed | *Clone 163* | CM000126 | Fang et al. (2009) | 0.85 | Up | 0.02 |
| 13 | LOC_Os01g43050 | Chr1:24559328-24568663 | CENP-C1, putative, expressed | *Clone 163* | CM000126 | Fang et al. (2009) | 0.85 | Up | 0.00 |
| 14 | LOC_Os01g46060 | Chr1:26183882-26190195 | NUC189 domain containing protein, expressed | *Clone 163* | CM000126 | Fang et al. (2009) | 0.74 | Up | 0.01 |
| 15 | LOC_Os01g48720 | Chr1:27938767-27947047 | RNA polymerase III subunit RPC82 family protein, putative, expressed | *Clone 163* | CM000126 | Fang et al. (2009) | 0.56 | Up | 0.03 |
| 16 | LOC_Os01g52110 | Chr1:29965063-29970767 | RING finger and CHY zinc finger domain-containing protein 1, putative, expressed | *Clone 163* | CM000126 | Fang et al. (2009) | -0.58 | Down | 0.02 |
| 17 | LOC_Os01g59100 | Chr1:34141402-34145203 | cytokinin-N-glucosyltransferase 1, putative, expressed | *Clone 512* | AP003734 | Fang et al. (2009) | -1.49 | Down | 0.01 |
| 18 | LOC_Os01g61380 | Chr1:35499016-35501765 | lactate/malate dehydrogenase, putative, expressed | *Clone 512* | AP003734 | Fang et al. (2009) | 0.61 | Up | 0.02 |
| 19 | LOC_Os01g62230 | Chr1:36013831-36014697 | Core histone H2A/H2B/H3/H4 domain containing protein, putative, expressed | *DEG-5* | CT828153.1 | Junaedi et al. (2008) | 0.54 | Up | 0.01 |
| 20 | LOC_Os01g62610 | Chr1:36253546-36256977 | peptidyl-prolyl cis-trans isomerase, FKBP-type, putative, expressed | *Clone 512* | AP003734 | Fang et al. (2009) | -1.00 | Down | 0.03 |
| 21 | LOC_Os01g63150 | Chr1:36587790-36600491 | retrotransposon protein, putative, Ty3-gypsy subclass, expressed | *Clone 265* | AC092263 | Fang et al. (2009) | -0.78 | Down | 0.04 |
| 22 | LOC_Os01g67740 | Chr1:39365919-39374228 | chromosome segregation protein, putative, expressed | *Clone 163* | CM000126 | Fang et al. (2009) | 0.75 | Up | 0.00 |
| 23 | LOC_Os01g70020 | Chr1:40501146-40507790 | DEK C terminal domain containing protein, expressed | *Clone 163* | CM000126 | Fang et al. (2009) | 0.48 | Up | 0.03 |
| 24 | LOC_Os01g71690 | Chr1:41539689-41545870 | recA protein, expressed | *Clone 163* | CM000126 | Fang et al. (2009) | 0.82 | Up | 0.02 |
| 25 | LOC_Os01g73160 | Chr1:42425423-42427258 | 40S ribosomal protein S10, putative, expressed | *Clone 593* | AP003263 | Fang et al. (2009) | 0.94 | Up | 0.00 |
| 26 | LOC_Os01g73250 | Chr1:42460554-42462334 | abscisic stress-ripening, putative, expressed | *Clone 593* | AP003263 | Fang et al. (2009) | -0.47 | Down | 0.04 |
| 27 | LOC_Os01g74030 | Chr1:42876538-42882964 | pumilio-family RNA binding protein, putative, expressed | *Clone 163* | CM000126 | Fang et al. (2009) | 0.54 | Up | 0.03 |
| 28 | LOC_Os02g02730 | Chr2:1029924-1034242 | expressed protein | *OsKSL4* | Os04g10060 | Xu et al. ( 2012) | -1.19 | Down | 0.00 |
| 29 | LOC_Os02g09140 | Chr2:4690517-4693590 | bfr-2, putative, expressed | *OsKSL4* | Os04g10060 | Xu et al. ( 2012) | 0.49 | Up | 0.03 |
| 30 | LOC_Os02g14929 | Chr2:8326082-8343178 | T-complex protein, putative, expressed | *Clone 587* | AP005578 | Fang et al. (2009) | 0.57 | Up | 0.03 |
| 31 | LOC_Os02g26850 | Chr2:15762104-15765880 | rRNA-processing protein FCF, putative, expressed | *DEG-2* | AY522330.1 | Junaedi et al. (2008) | 0.65 | Up | 0.04 |
| 32 | LOC_Os02g31160 | Chr2:18658027-18661837 | transcription factor, putative, expressed | *OsCPS4* | Os04g09900 | Xu et al. ( 2012) | -1.06 | Down | 0.01 |
| 33 | LOC_Os02g56990 | Chr2:34918110-34920148 | ribosomal protein L37, putative, expressed | *OsKSL4* | Os04g10060 | Xu et al. ( 2012) | 0.56 | Up | 0.02 |
| 34 | LOC_Os03g09880 | Chr3:4913121-4914145 | AIR12, putative, expressed | *OsKSL4* | Os04g10060 | Xu et al. ( 2012) | -0.75 | Down | 0.03 |
| 35 | LOC_Os03g11400 | Chr3:5865075-5869280 | targeting protein-related, putative, expressed | *Clone 512* | AP003734 | Fang et al. (2009) | 1.05 | Up | 0.00 |
| 36 | LOC_Os03g14530 | Chr3:7884814-7886832 | S10/S20 domain containing ribosomal protein, putative, expressed | *OsKSL4* | Os04g10060 | Xu et al. ( 2012) | 0.58 | Up | 0.01 |
| 37 | LOC_Os03g22340 | Chr3:12798127-12799750 | 60S ribosomal protein L22-2, putative, expressed | *Clone 188 (V)* | NM_001056613 | Song et al. (2008) | 0.56 | Up | 0.01 |
| 38 | LOC_Os03g22350 | Chr3:12802140-12805154 | Brix domain containing protein, putative, expressed | *OsKSL4* | Os04g10060 | Xu et al. ( 2012) | 0.61 | Up | 0.02 |
| 39 | LOC_Os03g22620 | Chr3:13031163-13046117 | terpene synthase family, metal binding domain containing protein, expressed | *Clone 512* | AP003734 | Fang et al. (2009) | 0.54 | Up | 0.05 |
| 40 | LOC_Os03g39610 | Chr3:21999941-22001714 | chlorophyll A-B binding protein, putative, expressed | *OsKSL4* | Os04g10060 | Xu et al. ( 2012) | 0.65 | Up | 0.01 |
| 41 | LOC_Os03g42220 | Chr3:23485490-23490741 | T-complex protein, putative, expressed | *OsKSL4* | Os04g10060 | Xu et al. ( 2012) | 0.55 | Up | 0.01 |
| 42 | LOC_Os03g43890 | Chr3:24621291-24629504 | WD domain, G-beta repeat domain containing protein, expressed | *OsKSL4* | Os04g10060 | Xu et al. ( 2012) | 0.54 | Up | 0.02 |
| 43 | LOC_Os03g45710 | Chr3:25800414-25801331 | 2Fe-2S iron-sulfur cluster binding domain containing protein, expressed | *DEG-2* | AY522330.1 | Junaedi et al. (2008) | -0.68 | Down | 0.00 |
| 44 | LOC_Os03g64170 | Chr3:36256014-36259499 | GDSL-like lipase/acylhydrolase, putative, expressed | *Clone 265* | AC092263 | Fang et al. (2009) | 0.47 | Up | 0.04 |
| 45 | LOC_Os03g64340 | Chr3:36366367-36367770 | heavy metal-associated domain containing protein, expressed | *Clone 265* | AC092263 | Fang et al. (2009) | 0.75 | Up | 0.02 |
| 46 | LOC_Os04g10060 | Chr4:5428337-5434028 | ent-kaurene synthase, chloroplast precursor, putative, expressed | *OsKSL4* | Os04g10060 | Xu et al. ( 2012) | 0.99 | Up | 0.02 |
| 47 | LOC_Os04g16764 | Chr4:9134186-9135280 | chloroplast 30S ribosomal protein S4, putative, expressed | *DEG-2* | AY522330.1 | Junaedi et al. (2008) | -1.06 | Down | 0.02 |
| 48 | LOC_Os04g16830 | Chr4:9190921-9195969 | DNA-directed RNA polymerase subunit beta, putative, expressed | *DEG-2* | AY522330.1 | Junaedi et al. (2008) | 0.87 | Up | 0.01 |
| 49 | LOC_Os04g25960 | Chr4:15098151-15106616 | expressed protein | *OsKSL4* | Os04g10060 | Xu et al. ( 2012) | 0.94 | Up | 0.04 |
| 50 | LOC_Os04g49194 | Chr4:29331562-29338265 | naringenin,2-oxoglutarate 3-dioxygenase, putative, expressed | *OsKSL4* | Os04g10060 | Xu et al. ( 2012) | -0.67 | Down | 0.02 |
| 51 | LOC_Os04g51520 | Chr4:30520355-30523556 | glycosyl hydrolases family 16, putative, expressed | *Clone 695* | AP003252 | Fang et al. (2009) | 1.35 | Up | 0.02 |
| 52 | LOC_Os04g59560 | Chr4:35422985-35426837 | tubulin folding cofactor B, putative, expressed | *Clone 695* | AP003252 | Fang et al. (2009) | 0.74 | Up | 0.01 |
| 53 | LOC_Os05g04890 | Chr5:2351572-2354876 | expressed protein | *OsKSL4* | Os04g10060 | Xu et al. ( 2012) | 1.01 | Up | 0.00 |
| 54 | LOC_Os05g35650 | Chr5:21171858-21191740 | peptide transporter PTR2, putative, expressed | *DEG-7* | AC122144.1 | Junaedi et al. (2008) | -0.70 | Down | 0.03 |
| 55 | LOC_Os05g42300 | Chr5:24738574-24742551 | NAF1 domain containing protein, expressed | *OsKSL4* | Os04g10060 | Xu et al. ( 2012) | 0.64 | Up | 0.03 |
| 56 | LOC_Os05g49860 | Chr5:28611972-28612798 | Core histone H2A/H2B/H3/H4 domain containing protein, putative, expressed | *DEG-5* | CT828153.1 | Junaedi et al. (2008) | 0.78 | Up | 0.00 |
| 57 | LOC_Os05g50610 | Chr5:29009135-29013172 | WRKY8, expressed | *Clone 265* | AC092263 | Fang et al. (2009) | -1.15 | Down | 0.00 |
| 58 | LOC_Os06g05250 | Chr6:2366601-2372121 | GTP-binding protein GUF1, putative, expressed | *OsKSL4* | Os04g10060 | Xu et al. ( 2012) | 0.51 | Up | 0.04 |
| 59 | LOC_Os06g11730 | Chr6:6208292-6214438 | RNA recognition motif containing protein, putative, expressed | *OsKSL4* | Os04g10060 | Xu et al. ( 2012) | 1.05 | Up | 0.00 |
| 60 | LOC_Os06g13670 | Chr6:7584779-7590605 | E2F family transcription factor protein, putative, expressed | *DEG-7* | AC122144.1 | Junaedi et al. (2008) | 0.97 | Up | 0.01 |
| 61 | LOC_Os06g28820 | Chr6:16400698-16432410 | cycloartenol synthase, putative, expressed | *Clone 695* | AP003252 | Fang et al. (2009) | -1.00 | Down | 0.01 |
| 62 | LOC_Os06g30320 | Chr6:17505124-17511961 | NOC3 - Putative nucleolar complex subunit 3, expressed | *OsKSL4* | Os04g10060 | Xu et al. ( 2012) | 0.64 | Up | 0.01 |
| 63 | LOC_Os06g37300 | Chr6:22020839-22028146 | cytochrome P450, putative, expressed | *OsCPS4* | Os04g09900 | Xu et al. ( 2012) | 1.26 | Up | 0.00 |
| 64 | LOC_Os06g46434 | Chr6:28180528-28181494 | cytochrome c biogenesis protein ccsA, putative, expressed | *DEG-2* | AY522330.1 | Junaedi et al. (2008) | -0.61 | Down | 0.05 |
| 65 | LOC_Os06g46435 | Chr6:28181690-28183193 | NADPH-dependent oxidoreductase, putative, expressed | *DEG-2* | AY522330.1 | Junaedi et al. (2008) | -0.60 | Down | 0.04 |
| 66 | LOC_Os07g22350 | Chr7:12544209-12550060 | glucose-6-phosphate 1-dehydrogenase, chloroplast precursor, putative, expressed | *OsKSL4* | Os04g10060 | Xu et al. ( 2012) | -0.52 | Down | 0.02 |
| 67 | LOC_Os08g38300 | Chr8:24261319-24262356 | Core histone H2A/H2B/H3/H4 domain containing protein, putative, expressed | *DEG-5* | CT828153.1 | Junaedi et al. (2008) | 0.74 | Up | 0.00 |
| 68 | LOC_Os08g41300 | Chr8:26090909-26092945 | 60S ribosomal protein L32, putative, expressed | *OsKSL4* | Os04g10060 | Xu et al. ( 2012) | 0.50 | Up | 0.03 |
| 69 | LOC_Os08g44850 | Chr8:28170357-28172330 | C2 domain containing protein, putative, expressed | *OsKSL4* | Os04g10060 | Xu et al. ( 2012) | -0.61 | Down | 0.01 |
| 70 | LOC_Os09g07450 | Chr9:3726391-3730896 | flavonol synthase, putative, expressed | *Clone 587* | AP005578 | Fang et al. (2009) | -0.81 | Down | 0.02 |
| 71 | LOC_Os09g07460 | Chr9:3732397-3734886 | kelch repeat protein, putative, expressed | *Clone 587* | AP005578 | Fang et al. (2009) | -0.53 | Down | 0.02 |
| 72 | LOC_Os09g16520 | Chr9:10131346-10138249 | cytochrome b5-like Heme/Steroid binding domain containing protein, expressed | *OsKSL4* | Os04g10060 | Xu et al. ( 2012) | 0.77 | Up | 0.02 |
| 73 | LOC_Os09g20684 | Chr9:12454364-12460996 | SMP-30/Gluconolaconase/LRE-like region containing protein, expressed | *Clone 512* | AP003734 | Fang et al. (2009) | -0.68 | Down | 0.01 |
| 74 | LOC_Os10g09870 | Chr10:5340965-5346935 | HVA22, putative, expressed | *Clone 512* | AP003734 | Fang et al. (2009) | 0.73 | Up | 0.01 |
| 75 | LOC_Os10g25660 | Chr10:13284008-13285673 | OsFBX385 - F-box domain containing protein, expressed | *DEG-7* | AC122144.1 | Junaedi et al. (2008) | 0.97 | Up | 0.04 |
| 76 | LOC_Os11g11020 | Chr11:6087978-6091482 | DAG protein, chloroplast precursor, putative, expressed | *DEG-2* | AY522330.1 | Junaedi et al. (2008) | 0.66 | Up | 0.03 |
| 77 | LOC_Os11g35090 | Chr11:20571983-20577400 | kinesin motor domain containing protein, putative, expressed | *Clone 512* | AP003734 | Fang et al. (2009) | 0.66 | Up | 0.03 |
| 78 | LOC_Os12g01730 | Chr12:434075-438686 | laccase-23 precursor, putative, expressed | *Clone 512* | AP003734 | Fang et al. (2009) | 0.77 | Up | 0.01 |
| 79 | LOC_Os12g05010 | Chr12:2176947-2178906 | retrotransposon protein, putative, unclassified, expressed | *Clone 265* | AC092263 | Fang et al. (2009) | 2.68 | Up | 0.03 |
| 80 | LOC_Os12g06980 | Chr12:3402726-3413832 | SAP domain containing protein, expressed | *Clone 163* | CM000126 | Fang et al. (2009) | 0.53 | Up | 0.02 |
| 81 | LOC_Os12g12950 | Chr12:7171809-7176578 | expressed protein | *OsKSL4* | Os04g10060 | Xu et al. ( 2012) | 1.11 | Up | 0.03 |
| 82 | LOC_Os12g19180 | Chr12:11137920-11141605 | ATP binding protein, putative, expressed | *DEG-2* | AY522330.1 | Junaedi et al. (2008) | 0.86 | Up | 0.00 |
| 83 | LOC_Os12g27090 | Chr12:15857978-15866742 | expressed protein | *Clone 512* | AP003734 | Fang et al. (2009) | 0.47 | Up | 0.04 |
| 84 | LOC_Os12g37510 | Chr12:23015818-23017657 | UDP-glucoronosyl and UDP-glucosyl transferase domain containing protein, expressed | *OsKSL4* | Os04g10060 | Xu et al. ( 2012) | -0.71 | Down | 0.01 |
| 85 | LOC_Os12g38620 | Chr12:23725966-23729784 | bromodomain associated family protein, expressed | *Clone 695* | AP003252 | Fang et al. (2009) | 0.50 | Up | 0.04 |
